# The conserved histone deacetylase Rpd3 and its DNA binding subunit Ume6 control dynamic transcript architecture during mitotic growth and meiotic development

**DOI:** 10.1093/nar/gku1185

**Published:** 2014-12-03

**Authors:** Aurélie Lardenois, Igor Stuparevic, Yuchen Liu, Michael J. Law, Emmanuelle Becker, Fatima Smagulova, Karl Waern, Marie-Hélène Guilleux, Joe Horecka, Angela Chu, Christine Kervarrec, Randy Strich, Mike Snyder, Ronald W. Davis, Lars M. Steinmetz, Michael Primig

**Affiliations:** 1 Inserm U1085-Irset, Université de Rennes 1, Rennes, F-35042, France; 2 School of Osteopathic Medicine, Rowan University, Stratford, NJ 08084, USA; 3 Department of Genetics, Stanford University, Stanford, CA 94395, USA; 4 Stanford Genome Technology Center, Palo Alto, CA 94304, USA; 5 Department of Biochemistry, Stanford University, Stanford, CA 94305, USA; 6 European Molecular Biology Laboratory, Heidelberg 69117, Germany

## Abstract

It was recently reported that the sizes of many mRNAs change when budding yeast cells exit mitosis and enter the meiotic differentiation pathway. These differences were attributed to length variations of their untranslated regions. The function of UTRs in protein translation is well established. However, the mechanism controlling the expression of distinct transcript isoforms during mitotic growth and meiotic development is unknown. In this study, we order developmentally regulated transcript isoforms according to their expression at specific stages during meiosis and gametogenesis, as compared to vegetative growth and starvation. We employ regulatory motif prediction, *in vivo* protein-DNA binding assays, genetic analyses and monitoring of epigenetic amino acid modification patterns to identify a novel role for Rpd3 and Ume6, two components of a histone deacetylase complex already known to repress early meiosis-specific genes in dividing cells, in mitotic repression of meiosis-specific transcript isoforms. Our findings classify developmental stage-specific early, middle and late meiotic transcript isoforms, and they point to a novel HDAC-dependent control mechanism for flexible transcript architecture during cell growth and differentiation. Since Rpd3 is highly conserved and ubiquitously expressed in many tissues, our results are likely relevant for development and disease in higher eukaryotes.

## INTRODUCTION

Meiosis is a developmental pathway that leads to the formation of haploid gametes. The process deviates from the mitotic cell cycle in several ways including extensive recombination and the execution of two nuclear divisions without an intervening S-phase (1,2). Previous studies identified genes that are repressed during vegetative growth, and specifically induced during early, middle and late stages of meiotic development (3–5).

Many members of the ‘early’ class of meiotic genes are transcriptionally repressed during mitosis by a conserved histone deacetylase (HDAC) complex including the deacetylase Rpd3, the co-repressor Sin3 and the DNA-binding protein Ume6, which recognizes an upstream regulatory site 1 (URS1) (6,7). RNA profiling experiments and genome-wide DNA-binding assays analysing mitosis and meiosis revealed numerous differentially expressed genes, among them are many that are directly regulated by Rpd3 and Ume6 (8–10). The Rpd3 core complex represses its targets by stabilizing nucleosomes, and by an activity independent of histone deacetylation (11). Rpd3/Sin3/Ume6-dependent repression is relieved through a two-step system targeting Ume6 for destruction. The first step occurs in cells switching from fermentation to respiration, which induces acetylation by the Spt-Ada-Gcn5-acetyltransferase (SAGA) complex resulting in partial Ume6 destruction by the anaphase promoting complex/cyclosome (APC/C). Final Ume6 destruction occurs once the cells enter meiosis and requires the meiotic inducer Ime1. Ultimately, Ume6 re-accumulates during late stages of spore formation when it plays an important role in germination (12–14).

It is well established that DNA binding regulators cooperate with chromatin modification enzymes to repress meiosis-specific genes during vegetative growth (3). However, it has only recently emerged that a whole class of genes encodes several isoforms that change in length—typically due to variable 5′- and 3′-untranslated regions (UTRs)—when yeast cells respond to stress (15,16), or when they exit mitosis and enter meiosis (17–19). Little is known about the transcriptional mechanisms governing this process. UTRs control mRNA stability, transport and translation through interaction with numerous RNA-binding proteins. Their flexible architecture has therefore broad implications for the regulation of protein expression during mitosis (20), filamentous growth (21) and developmental pathways, such as meiosis and gametogenesis (22–24). A well-studied mechanism of 5′-UTR-mediated translational control acts via upstream open reading frames (uORFs), which were recently reported to positively or negatively regulate translation in sporulating budding yeast cells (25,26).

The budding yeast transcriptome of mitotic growth and meiotic differentiation has been the focus of numerous studies based upon microarrays typically covering entire genes or their 3′-regions (4,5), tiling arrays covering the complete genome on both strands (27,28) and RNA-sequencing (RNA-Seq). This recent method for RNA profiling employs ultra high-throughput DNA sequencing (15,19,29). As genomics technology improved, it became feasible to determine transcript-splicing patterns, and to identify multiple transcript isoforms from single genes (15,20,30).

High-throughput DNA sequencing and RNA profiling technologies spawned the development of bioinformatics tools useful for finding biologically relevant regulatory motifs. The TRANSFAC database provides information on DNA binding transcription factor (TF) target sites represented by position weight matrices (PWMs), which help gain insight into the regulatory composition of promoters (9,10,31–34). A PWM is built by aligning the sequences of all known binding sites a given TF interacts with, and log-transforming the number of observations of each nucleotide at each position (35,36). This method is therefore employed to predict a range of sequence motifs that likely interact with DNA binding TFs of interest, such as Ume6 (for more details, see the ScerTF database at http://stormo.wustl.edu/ScerTF (37)).

The present study is designed to identify, classify and validate developmentally regulated isoforms showing increased UTR length, and, importantly, to gain insight into the transcriptional mechanism that controls meiosis-specific isoforms containing extended 5′-UTRs. We investigated RNA concentration and transcript architecture in a comprehensive sample set comprising synchronously growing haploid *MAT***a** cells, fermenting or respiring *MAT***a**/α cells, a starving meiosis-deficient *MAT*α/α strain and sporulating *MAT***a**/α cells (27,28). Our genomic analysis is based on tiling arrays covering both strands of the entire yeast genome, and was confirmed using RNA-Seq data (Becker *et al.*, in preparation), and molecular biological methods. We found that predicted URS1 motifs bound by Ume6 are statistically significantly enriched in promoters that mediate the expression of distinct transcript isoforms. This led to our discovery of a direct role for the conserved HDAC Rpd3 and its DNA binding interactor Ume6 in repressing meiotic transcript isoforms during vegetative growth.

## MATERIALS AND METHODS

### Yeast strains, media and sporulation protocols

The tiling array data were produced with diploid SK1 *MAT***a**/α and *MAT*α/α strains and validation experiments were done in SK1 *MAT***a**/α, JHY222 *MAT***a**/α strains as published (28). mUTR expression was assayed in SK1 *MAT***a**/α *ume6* (see (8)) and *rpd3*, and JHY222 *MAT***a**/α *ume6* deletion strains. Sporulation experiments were done in rich medium with glucose (YPD) or acetate (YPA) and sporulation medium (SPII); sporulation was verified by monitoring pre-meiotic DNA replication, and by counting bi- and tetranuclear cells, and asci using standard procedures (Supplementary Additional Table 1) (28).

### Construction of C-terminally tagged proteins

A polymerase chain reaction (PCR)-based one-step tagging method was used to generate strains expressing the C-terminally tagged proteins using cassette plasmids and oligonucleotides as described (38,39). Colonies were first screened by PCR for correct integration and then validated by western blotting. Oligonucleotides used are shown in Supplementary Additional Table 2.

### URS1 deletion using the 50:50 method

The 50:50 method for PCR-based seamless genome editing in yeast was done as described (40). Briefly, to delete the URS1 motif in the *RTT10* promoter we first transformed haploid JHY336 *MAT***a** and JHY337 *MAT*α cells with a PCR fragment to insert the *URA3* marker into the target site (*RTT10* 50/50-U2 and D2-50 *RTT10*, Supplementary Additional Table 3). *URA3*^+^ colonies harbouring the intended insertion event were identified by diagnostic PCR, and spread onto solid synthetic complete medium containing 5-Fluoroorotic acid (5-FOA; Euromedex, France) to select for loss of *URA3* and URS1. Several *ura3* mutant colonies were analysed by diagnostic PCR, and the URS1 deletion was confirmed by sequencing genomic DNA. Haploid *MAT***a** urs1Δ and *MAT*α urs1Δ cells were mated to yield a *MAT***a**/α urs1Δ/urs1Δ strain.

### RNA isolation and microarray raw data production

The molecular biological methods and approaches used for raw data processing and normalization are described in (28).

### mUTR selection procedure

**Figure 1.**
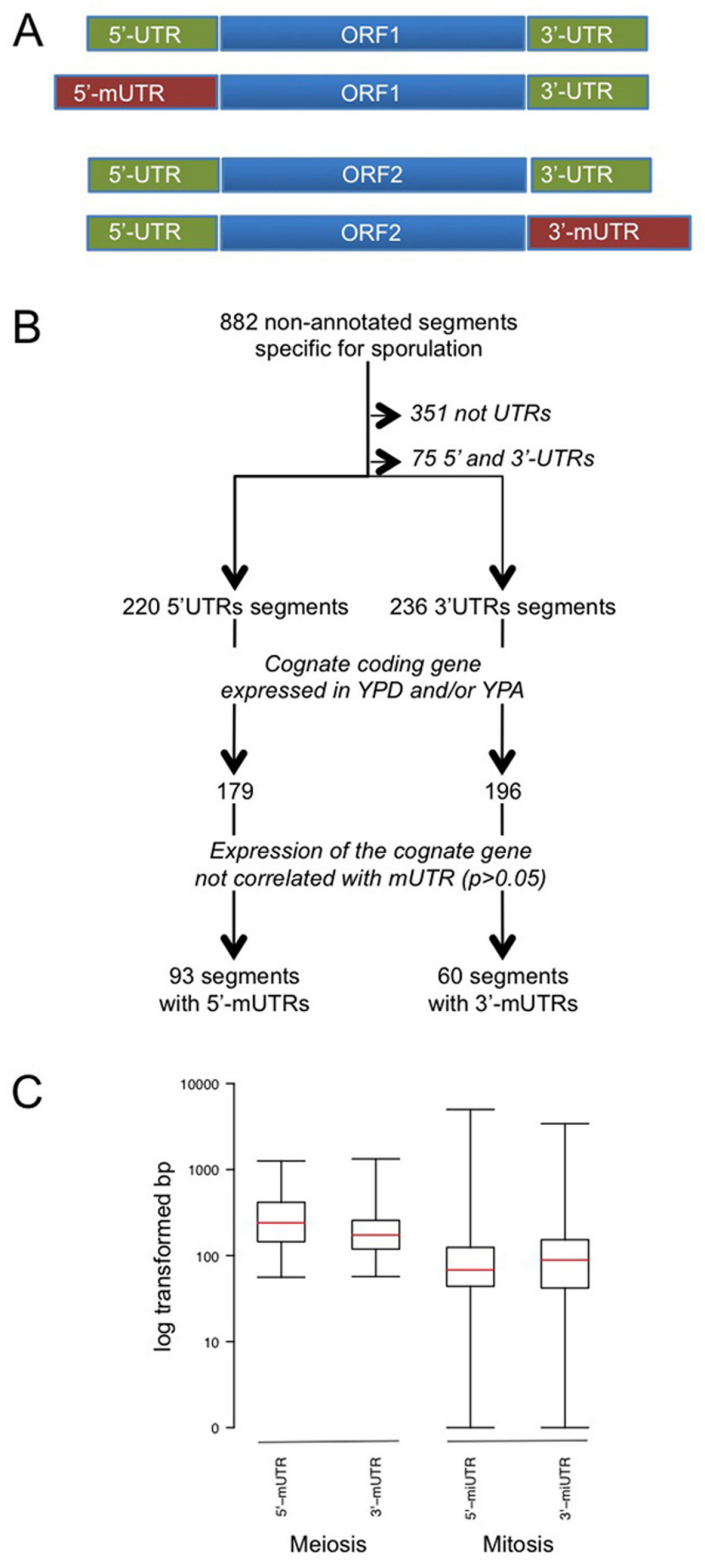
Definition, identification and characterization of mUTRs. (A) The schematic shows two prototype genes (ORF1 and ORF2 shown in blue, UTRs shown in green) encoding transcript isoforms with extended 5′-meiotic UTRs (5′-mUTRs) or 3′-meiotic UTRs (3′-mUTRs), respectively (shown in red). (B) A flow chart depicts the key steps of the mUTR annotation procedure and its output. (C) UTR length distribution in mitosis and meiosis. A box plot shows the size distribution in log-transformed lengths (in bp; *y*-axis) for 5′-UTRs and 3′-UTRs during meiosis and mitosis (*x*-axis). The red line is the median.

In previous work we employed a segmentation algorithm to analyse yeast transcriptome using data obtained from growing, sporulating and starving cells (28). To identify mRNAs with changing 5′- and 3′-UTRs we first selected 882 segments that were not annotated as known protein-coding genes, snoRNAs, rRNAs and tRNAs, stable unannotated transcripts (SUTs), cryptic unstable transcripts (CUTs) or meiotic unannotated transcripts (MUTs), and for which expression signals above background were detected only in sporulating cells (Figure 1). To decrease the likelihood of inadvertently annotating independent transcripts as UTRs, only segments located <100 base pairs (bp) from a transcription start site (TSS) or the terminator of an ORF were considered to be potential 5′- and 3′-UTRs, respectively. We selected mUTRs of protein-coding genes expressed in fermenting cells (YPD), respiring cells (YPA) or in both conditions. Expression correlations were estimated based on averaged expression data from YPD, YPA and SPII (sporulation) samples using Pearson's product moment correlation coefficient (cc), which follows a *t*-distribution with 12 degrees of freedom. The resulting *P*-values were adjusted with the false discovery rate procedure of Benjamini and Hochberg (41). Segments showing no significant expression correlation with their adjacent ORFs (*P*-value > 0.05) were selected as 5′- and 3′-mUTRs (Figure 1B). This method yielded a representative set of mUTRs but was unable to identify certain cases showing complex segmentation patterns or ambiguous annotation of ORFs and lncRNAs. Such issues cannot be resolved with tiling array data alone, and will therefore require information provided by future DNA strand-specific analyses of the meiotic transcriptome by RNA-Seq and molecular validation experiments.

### Regulatory motif enrichment analysis

The average size of a yeast promoter is ∼300 bases and the median of 5′-mUTR size distribution is 241 bases. We therefore investigated the enrichment of predicted URS1 sites in a region restricted to 100 bases upstream of 5′-mUTRs, using PWMs M01503 and M01898 provided by the TRANSFAC Professional database release 2011.4 (42). The Promoter Analysis Protocol (PAP) (43) was executed using the TRANSFAC minSUM profile. Predicted URS1 motifs were filtered using a minCSS (core score similarity) cut-off set at 0.9, and a minMSS (matrix score similarity) cut-off set at 0.7. In addition, motif enrichment was estimated using the algorithms implemented in MEME (44), DREME (45), Tomtom (46) and Clover (47) software. In the case of Clover, enrichment was estimated as compared to regions of 100 bp upstream of the cognate protein-coding genes. We considered motifs to be enriched at a *P*-value ≤ 0.05 when using PAP, MEME and DREME, and at a *P*-value ≤ 0.01 in the case of Clover.

### Northern blotting analysis

400 ng of poly*A*^+^ RNA was run on a 1% agarose gel (2% formaldehyde, 0.2 M MOPS, 0.05 M sodium acetate, 0.01 M ethylenediaminetetraacetic acid (EDTA); Sigma, France) for 5 h at 100 V. The gel was rinsed in H_2_O and saline sodium citrate (10× SCC; 3 M sodium citrate, 0.3 M sodium chloride; Sigma, France) before the RNA was transferred to nylon HYBOND N (Amersham, UK) membrane overnight in 10× SCC. The membrane was exposed to ultraviolet light (Stratalinker, USA), stained with methylene blue and stored at −20°C until further use. DNA probes were synthesized using the RadPrime kit (Invitrogen, USA). 60 ng of PCR-amplified DNA was incubated at 95°C for 5 min and 4°C for 5 min, mixed with 20 μl of buffer, 3 μl of dATG mix (500 μM each), 5 μl of α-P^32^-dCTP (50 μCi) (Amersham, UK) and 1 μl (40U) Klenow enzyme (Boehringer, USA), and incubated at 37°C for 30 min. Labelled DNA was precipitated and its specific activity was determined with a Bioscan Counter (Packard, USA) in 3 ml of scintillation buffer (Ultima-Gold-MV, USA). The membrane was pre-hybridized at 65°C and hybridized overnight in 25 ml buffer (6× SSC, 5× Denhardt's, 10 mM PO_4_ buffer, 0.5% sodium dodecyl sulphate (SDS)) containing 500 μl salmon sperm DNA (7 mg/ml) and 200 μl of radioactive probe denatured at 100°C for 10 min and 5 min at 4°C. The membrane was washed in 1× SCC, 0.1% SDS at 65°C three times for 15 min and exposed to a Storage Phosphor Screen for 30 h. Data were produced with the Storm Imager 825 (General Electric, USA) at the default settings. Image files were processed and band intensities were computed using *ACT1* as an internal control with the ImageQuantTL 7.0 software (General Electric, USA). Primer sequences are shown in Supplementary Additional Table 4.

### 5′-Rapid Amplification of cDNA Ends (5′-RACE) analysis

5′ RACE was performed using the 5′-RACE version 2.0 kit (Invitrogen) following the manufacturer's protocol. RNA integrity was verified by gel electrophoresis and the concentration was determined using a Nanodrop spectrophotometer (Thermo Scientific). First strand cDNA was synthesized using a gene-specific primer (GSP1) and SuperScript II reverse transcriptase, and the mRNA template was removed by treatment with RNase H (specific for RNA:DNA heteroduplex molecules) and RNase T1. Unincorporated dNTPs, GSP1 and proteins are separated from cDNA using a S.N.A.P.™ Column. A homopolymeric tail was added to the 3′-end of the cDNA using Terminal deoxynucleotidyl Transferase and dCTP. DNA amplification was carried out with GSP2 and UAP (Universal Amplification Primer). The PCR products of the expected size were purified from the agarose gel and sequenced. The sequences of primers used in 5′-RACE assays are provided in Supplementary Additional Table 5.

### Reverse transcriptase-PCR (RT-PCR) assays

To design RT-PCR oligonucleotide primers using the Primer3 online tool (simgene.com/Primer3; (48)) we retrieved the target gene ORFs from Saccharomyces Genome Database (SGD; yeastgenome.org), and then aligned it with the SK1 genome using the online *Saccharomyces Genome Resequencing Project* Browser software (sanger.ac.uk/research/projects/genomeinformatics/sgrp.html; (49)). Similarly, DNA sequences corresponding to newly annotated mUTRs were retrieved from SGD based on their genome coordinates and aligned to the SK1 genome. RT-PCR reactions were done with 2 μg of RNA that was reverse transcribed with RT (High Capacity cDNA Reverse Transcription kit; Life Technologies, USA) and amplified using Taq Polymerase (Qiagen, France) at 60ºC for 26 cycles. Samples were run on 2% agarose gels and photographed using an ImageQuant 350 digital Imaging System at the default settings (General Electric, USA). Primer sequences are shown in Supplementary Additional Table 6.

### Western blotting analysis

Samples for protein analysis were prepared and analysed from fermenting, respiring and sporulating cells as published (28). Note that 25 μg of total protein extract was run on a 4–20% gradient gel (BioRad, USA) for 1 h. Proteins were transferred onto ImmobilonPSQ membranes (Millipore, France) using an electro-blotter system (TE77X; Hoefer, USA) and a modified Towbin buffer (48 mM Tris base, 40 mM glycine and 0.1% SDS) and methanol (20% vol/vol anode; 5% vol/vol cathode) for 2 h. Proteins were detected using a monoclonal anti-myc-horseradish peroxidase antibody (Life Technologies, USA) at 1:1000. The antibodies were incubated in hybridization buffer overnight. The signals were revealed using the ECL-Plus Chemiluminescence kit (GE Healthcare, USA) and the ImageQuant 350 system (GE Healthcare, USA). Band intensities were normalized and quantified using the ImageQuant TL 7.0 software and default parameters. A polyclonal antibody against Pgk1 (Invitrogen, USA) was employed as a loading control.

### Chromatin immunoprecipitation (ChIP) assay

The Ume6 protein bound to DNA *in vivo* was precipitated using a standard assay with cells cultured in rich media (YPD, YPA) and sporulation medium (SPII, 4, 8 and 10 h). Briefly, we analysed an SK1 strain expressing native Ume6 protein as described previously with a number of modifications (50). 20 ml of SPII cultures or 50 ml of mid-log YPA cultures were cross-linked with 1% formaldehyde for 15 min at room temperature. Cross-linked protein/DNA complexes were quenched with 140 mM glycine (Sigma, USA) for 5 min. Anti-Ume6 immune complexes were collected, washed and eluted prior to reversing crosslinks. DNA was precipitated, treated with proteinase K and amplified by Q-PCR as published (12). Relative ChIP signals were calculated using the formula (2^∧^-IP (CT target – CT control)/input (CT target – CT control)). The *NUP85* coding region was used as an internal control (Supplementary Additional Table 7). To study lysine acetylation *in vivo* we compared duplicate wild-type (WT) and *rpd3* mutant samples grown in rich medium (YPD) using a polyclonal antibody directed against acetylated lysine residues present in proteins (Abcam, USA; ab21623). Data were normalized as follows: standard curves were done for each primer set enrichment relative to a control without antibody and the WT data were set at 1 to visualize the fold-changes observed in the *rpd3* mutant. *SPO13* was used as a positive control using oligonucleotide primers as published (51). To analyse the protein–DNA interaction of RNA polymerase II we used a monoclonal antibody against its largest subunit Rpo21/Rpb1 (Covance, France). 40 ml of logarithmically growing yeast cells were fixed for 15 min in 1% formaldehyde (Sigma, USA). The reaction was stopped by adding 2.7 ml 2.5M glycine and after the cells were washed in 1× TBS (1 M Tris pH 7.5, 5 M NaCl) and lysis buffer (1% SDS, 10 mM EDTA, 50 mM Tris pH 8 with complete protein inhibitor cocktail (Roche, USA)). The cells were vortexed 10 times for 2 min and kept on ice for 2 min between each cycle. The samples were centrifuged and the chromatin in the supernatant was sheared using 15 cycles (10 s on, 30 s off at medium level) with a BiogenodeTM UCD200 sonicator (Diagenode, Belgium). Next, the samples were incubated with the anti-Rpo21/Rpb1 antibody and protein A coupled to Dynabeads (Life Technologies, USA) overnight at 4°C. The beads were washed as described (52). Chromatin was eluted with 1% SDS, 0.1 M NaHCO_3_ pH 9 at 65°C and crosslinking was reversed at 65°C overnight. The DNA was treated with a proteinase K (New England Biolabs, France) for 2 h at 45°C. Samples were purified with a MinElute Reaction Clean up kit (Qiagen, France). The DNA concentration was determined with a Quantus Fluorometer (Promega, USA) using the QuantiFluor dsDNA System Kit (Promega). Real-time PCR was done using 0.5 ng DNA and the GoTaq qPCR Master Mix Kit (Promega). Reactions were performed in a 7500 Real-Time PCR machine (Life Technologies, USA) according to the manufacturer's instructions. The gene copy number was calculated with 7500 Software version 2.0.5 (Life Technologies, USA). The fold-enrichment was calculated as the ratio of the precipitated gene copy number versus the input normalized over a negative control (RDN25-1). *ACT1* was used as a positive control. DNA fold-enrichment was also evaluated by standard PCR analysis using the AmpTaq Gold 360 Master Mix Kit (Life Technologies, USA). To amplify DNA within the linear range 27 cycles were run at 95°C for 15 s, followed by 60°C for 20 s and 70°C for 20 s (Supplementary Additional Table 8).

## RESULTS

### A subset of genes expresses developmental stage-specific transcript isoforms that increase in length

Using information on segment (transcript) annotation from our previously published tiling array data set, we identified constitutively expressed mRNAs for which putative 5′- or 3′-UTRs increase in length as cells undergo meiotic differentiation (28). Such cases are classified as transcripts possessing an extended meiotic UTR (mUTR; Figure 1A). By employing an automated approach we selected a representative, but not exhaustive, set of 93 segments annotated as potential 5′-mUTRs (corresponding to 92 genes). In addition, 60 segments were chosen corresponding to as many predicted 3′-mUTRs that increase in size during sporulation (Figure 1B). Our method's output concerning 5′-mUTRs complements a recent transcript and ribosome profiling study based on RNA-Seq; the data in this study confirms 23 of our 92 5′-mUTRs (22%; Supplementary Additional file 1) (26). This fairly small overlap is likely due to different RNA profiling technologies and distinct approaches used to identify transcripts with extended 5′-UTRs.

Tiling array data and RNA-Seq data alone do not prove that a given gene encodes distinct transcript isoforms that cover the entire locus. However, in light of our own validation work and results published by others, it is plausible to speculate that many yeast genes encode *bona fide* transcript isoforms with variable 5′ and 3′ regions (15,19,20,26,29,53). While it has been shown before that certain yeast genes encode long meiotic transcript isoforms not expressed in starving cells (19,26), this study focussed on the unanswered questions of developmental stage-specific mUTR timing, and, importantly, the transcriptional regulation of mRNAs with flexible 5′-mUTRs.

The median length of 5′-mUTRs selected using the procedure shown in Figure 1C was determined to be 241 bp, ranging from 56 to 1257 bp. We found the 3′-mUTRs to be somewhat shorter with a median of 173 bp, ranging from 57 to 1329 bp. This result revealed a 3.5-fold size increase (*P*-value for Wilcoxon signed-rank test <10^–15^) as compared to previously published mitotic 5′-UTRs (median length of 68 bp) and a 1.9-fold increase (*P*-value < 10^–10^) as compared to mitotic 3′-UTR (91 bp) that were determined by tiling arrays (53).

Our current analysis did not yield any significantly enriched Gene Ontology terms among the genes referred to in Figure 1B (54). This result may change as yeast genome annotation (hence our ability to detect biologically relevant mUTRs) improves. In any case, it is noteworthy that we identified genes involved in processes relevant for meiosis and gametogenesis. This includes loci conserved from yeast to human, and covers for example mitochondrial biogenesis and function (*ATP18*, *FMP27*, *MBA1*, *MRPL27*, *RPO41*), DNA replication (*MCM5*), DNA repair (*RAD2*, *MLH2*), sister chromatid cohesion (*PDS5*), cytokinesis (*SHS1*), chromatin modification (*ARD1*, *IES1*), RNA processing (*CFT2*, *UTP6*), protein catabolism (*PUP2*), as well as transport and metabolism (*GOS1*, *MNN1*, *MUP1*, *MUP3*, *RTT10*, *XKS1*, *YPT11*).

### Promoters expressing early meiotic isoforms contain the Ume6 target motif URS1

**Figure 2.**
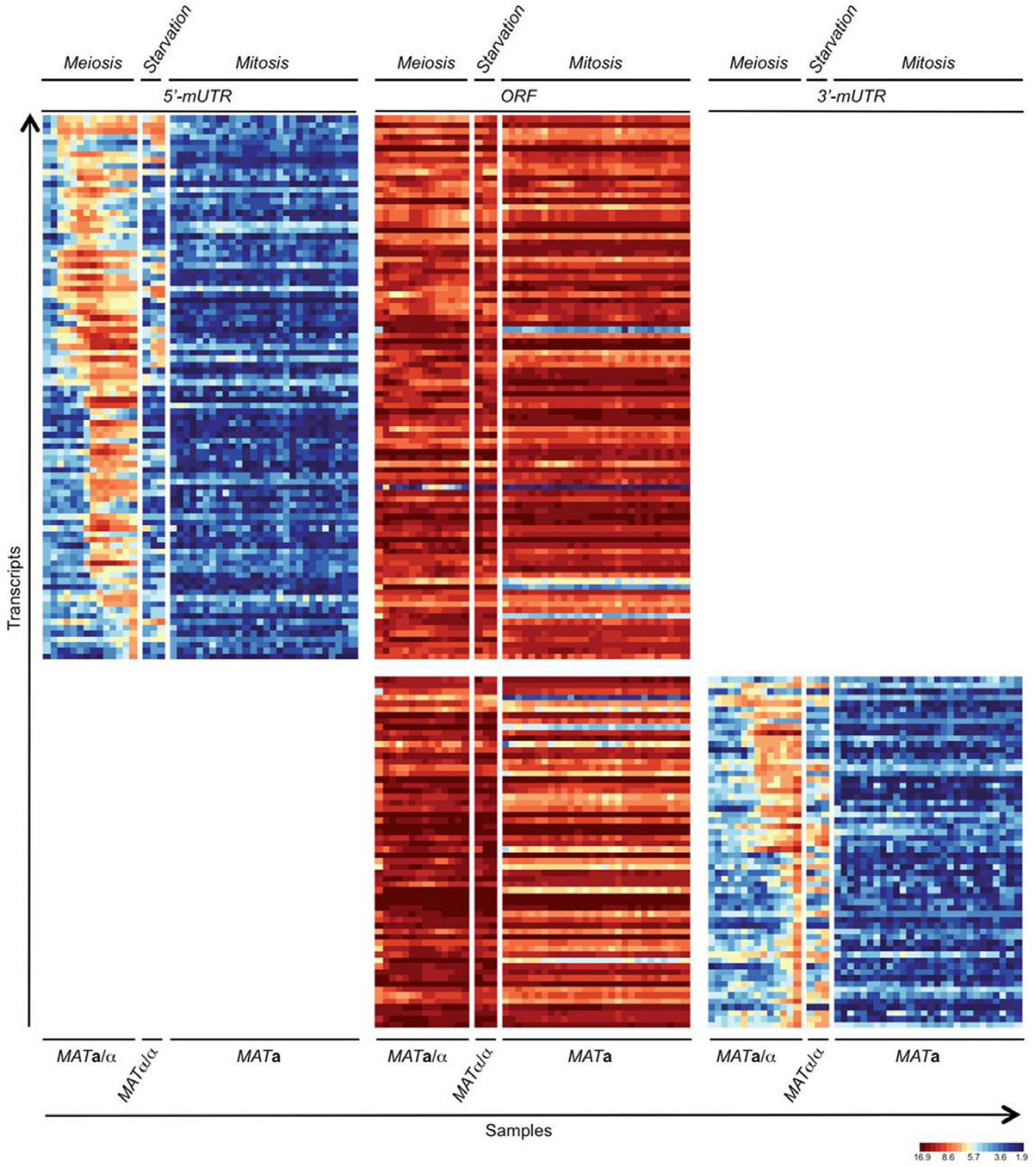
Transcript isoform expression during mitosis, meiosis and starvation. A false-colour heatmap is shown for transcript 5′-mUTRs (left column), ORFs (middle column) and 3′-mUTRs (right column). Samples from haploid (*MAT***a**), diploid (*MAT***a**/α) and diploid sporulation-deficient (*MAT*α/α) strains are indicated at the bottom. *MAT***a**/α cells were harvested in YPD, YPA and SPII 1–12 (hourly time points; Meiosis). *MAT*α/α samples were from cells cultured in YPA, SPII 8 and 10 h (Starvation). *MAT***a** cells were sampled every 5 min following α-factor synchronization between 0 and 135 min (Mitosis). Each line on the *y*-axis corresponds to a transcript and each column on the *x*-axis represents a set of samples as indicated. A colour scale for normalized and log2-transformed expression signals is given.

In our data set, we were able to select 5′- and 3′-mUTRs that reiterate the staggered induction pattern previously observed for early, middle and mid-late meiotic protein-coding transcripts (55). Moreover, we observed that these mUTRs are typically neither expressed in a haploid *MAT***a** strain undergoing mitotic cell cycles, nor in fermenting or respiring diploid *MAT***a**/α cells, nor in starving *MAT*α/α controls. In contrast, we detected transcripts corresponding to the protein-coding regions under all experimental conditions (Figure 2). These results are consistent with and extend previous reports of transcript isoforms expressed during gametogenesis (19,26). Next, we confirmed the tiling array data using information from an RNA-Seq experiment, which compares the transcriptome of *MAT***a**/α cells undergoing fermentation, respiration and sporulation to a fermenting, respiring and starving *MAT*α/α control (Supplementary Additional file 2; the complete data set will be published elsewhere; E. Becker *et al.*, in preparation).

Many of the transient 5′-mUTR expression patterns were broadly reminiscent of early meiotic genes that are repressed by the Ume6/Rpd3/Sin3 complex in mitosis. Thus, we asked if the Ume6 DNA binding target motif URS1 was enriched in the immediate upstream regions of our target genes. To this end, we used the Promoter Analysis Protocol (see Materials and Methods) based on two PWMs (M01503, M01898) for URS1 provided by the TRANSFAC database (34). This approach complements and vastly extends information about the classical URS1 motif reported 18 years ago (56). We found that the algorithm implemented in Clover identified predicted URS1 motifs as being significantly enriched (M01503 *P*-value 0.001, M01898 *P*-value 0.008; see Materials and Methods). A total of 21 to 61 out of 92 UTRs (23–66%) contain a URS1 depending on how stringent the filtration criteria were set (Supplementary Additional file 1, see Materials and Methods). The fact that a substantial fraction of the 5′-mUTRs lack URS1 motifs predicted with high confidence is consistent with our finding that mUTRs fall into all three expression classes (early, middle and late) with the final two typically being controlled by Ume6-independent regulatory systems.

### Meiotic isoforms are repressed by Rpd3/Ume6 during vegetative growth

**Figure 3.**
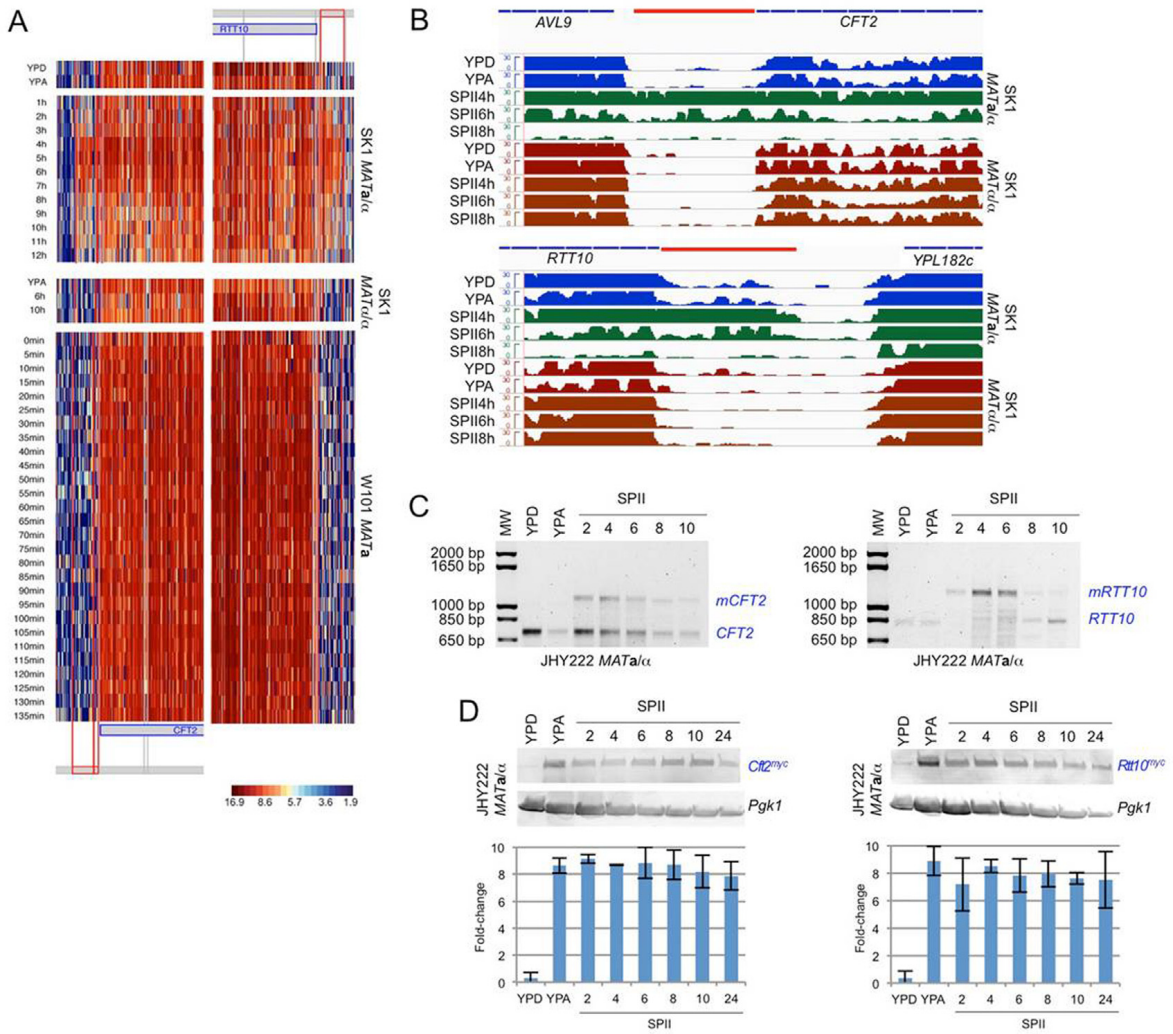
*RTT10* and *CFT2* early meiosis-specific isoform expression. (A) Heatmaps are shown for 5′-regions of *CFT2* and *RRT10* that contain predicted URS1 motifs. Blue rectangles are ORFs. Segments corresponding to the extended 5'-UTRs are shown in red. The raw output of the segmentation algorithm is shown in grey. Samples and strains are indicated. The log2 scale is given. (B) Histograms representing RNA-Seq data (not DNA strand specific) obtained with diploid SK1 WT and control strains are shown. Data on reads are presented on a linear scale (*y*-axis). RNA was isolated from fermenting (YPD), respiring (YPA) and sporulating (SPII 4, 6, 8 h) cells as indicated. Mitotic samples from the WT strain are given in blue, meiotic samples are given in green. Samples from the control strain are shown in red. Thin blue lines represent the ORFs, and a red line represents the 5′-mUTR extension (based on DNA strand-specific tiling array data). (C) 5′-RACE data are shown for *CFT2* and *RTT10* isoforms as indicated. Molecular weight markers (MW) are given in bp. Cells were cultured in rich medium (YPD) or sporulation medium (SPII, 2, 4, 6, 8 and 10 h). (D) A western blot is shown for Cft2 and Rtt10 containing a C-terminal myc tag (Cft2^myc^, Rtt10^myc^) in rich media (YPD, YPA) and sporulation medium at the time points indicated (SPII). Pgk1 was used as a loading control. Histograms show the fold-change of signal intensities determined in triplicate relative to the loading control (*y*-axis) versus the samples (*x*-axis); error bars of the SD are given.

We validated the 5′-mUTRs of *CFT2* (mRNA cleavage and polyadenlylation) and *RTT10* (endosomal recycling) because these conserved genes encode transcripts with extended 5′-mUTRs that are induced during early meiosis but not vegetative growth, or starvation, according to tiling array signals (Figure 3A) and RNA-Seq data (Figure 3B) (26). To determine the abundance of the mitotic and meiotic isoforms during growth and development we analysed *CFT2* and *RTT10* by 5′-RACE experiments using RNA from fermenting (YPD), respiring (YPA) and sporulating (SPII) cells. We found both mitotic and meiotic isoforms for *CFT2* in sporulating cells while *RRT10* mostly expresses the long isoform when cells undergo meiotic development. Consistently, expression signals decrease as cells exit M-phase and enter spore formation (Figure 3C). We next constructed strains expressing Cft2 and Rtt10 proteins with C-terminal myc tags that displayed no discernible growth or sporulation phenotype. For technical reasons we used JHY222, which is derived from a commonly used genetic background (S288C), and was engineered to sporulate almost as well (albeit not as fast) as SK1 (28). A western blot analysis of triplicate samples from cells cultured in rich media (YPD, YPA) and sporulation medium (SPII) revealed an ∼8-fold increase of protein levels in respiring as compared to fermenting cells, with peak levels being reached during meiotic M-phase and late spore maturation (a representative blot is shown in Figure 3D). Taken together, these results are consistent with a regulatory mechanism exerting its effect at the level of protein stability or protein translation (or both) via carbon-sources and metabolic processes.

**Figure 4.**
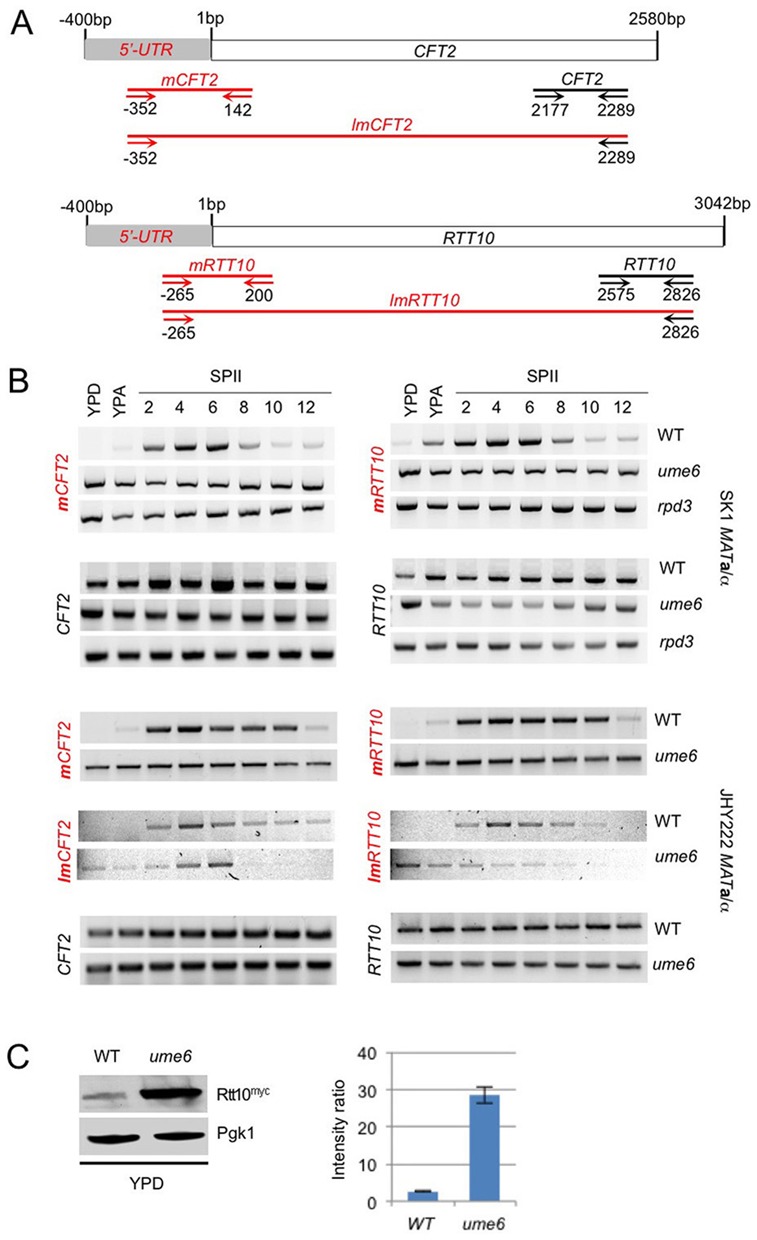
Early meiotic isoform expression in *rpd3* and *ume6* mutants. (A) A schematic shows 5′-UTRs and ORFs as grey and white rectangles. Sequence length is indicated in bp. Diagnostic PCR fragments are shown as red and black lines covering meiotic and mitotic isoforms, respectively. Small arrows represent forward and reverse PCR primers for which the coordinates are given in bp. To assay the meiotic isoforms we used two primer combinations that reveal mUTRs and cover short (*mCFT2*, *mRTT10*) or long (*lmCFT2*, *lmRTT10*) ORF regions as indicated. (B) The output of RT-PCR assays for SK1 WT and mutant (*ume6*, *rpd3*) strains, and JHY222 WT and *ume6* strains is shown for mitotic transcripts (given in black) and meiotic isoforms detected with two primer pairs (red). (C) A western blot is shown for myc-tagged Rtt10 (Rtt10^myc^) detected in duplicate samples from WT and Ume6 deletion (*ume6*) strains cultured in rich medium (YPD). Pgk1 was used as a loading control. A histogram is shown as in Figure 3D.

We then explored the biological relevance of predicted URS1 motifs in the target gene promoters. First, we further examined target transcript architecture by RT-PCR using primers that detect the mitotic isoform (*CFT2*, *RTT10*), and two combinations of primer pairs that reveal the meiotic isoform in both SK1 and JHY222 WT strains (*mCFT2*/*lmCFT2* and *mRTT10*/*lmRTT10*; Figure 4A and B; quantified band intensities are shown for mUTRs in Supplementary Additional file 3). RT-PCR data reproduced the expression patterns observed with tiling arrays; the weak PCR signal we detect in respiring cells (YPA) might be due to the assay's high sensitivity, promoter leakage or premature entry into meiosis of a small fraction of cells in YPA pre-sporulation medium.

Next, we monitored the expression of meiotic isoforms during vegetative growth in the absence of a functional Rpd3/Ume6 repressor complex. SK1 cells lacking Ume6 or Rpd3, and JHY222 cells lacking Ume6 strongly expressed transcripts including the extended *CFT2* and *RTT10* 5′-mUTRs during vegetative growth in rich media containing glucose or acetate. Moreover, Ume6 and Rpd3 mutants continued to express the early meiotic isoforms until spore maturation (Figure 4B). Control primers probing the *CFT2* and *RTT10* ORFs yielded homogenous signals across all samples in both backgrounds (Figure 4B). Weak signals were observed for *lmCFT2* and *lmRTT10* in JHY222 WT and *ume6* cells because large fragments amplify less well than small ones using the standard PCR parameters we applied to all assays. We subsequently asked if the de-repression of *RTT10*'s long meiotic isoform during rapid mitotic growth in *ume6* mutant cells coincides with a substantial increase in the Rtt10 protein level, and found this indeed to be the case (Figure 4C).

**Figure 5.**
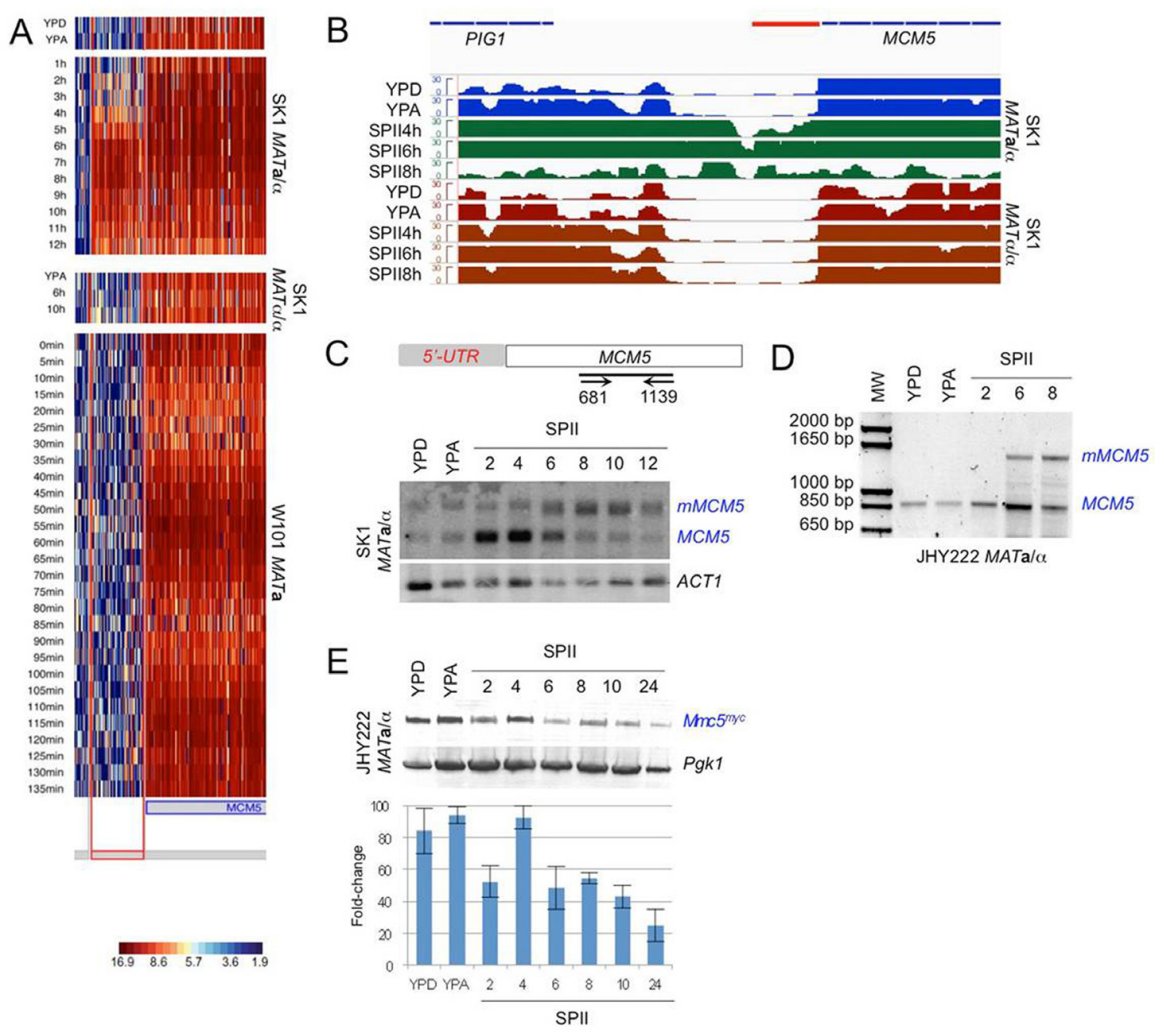
*MCM5* middle meiosis-specific isoform expression. (A and B) Heatmaps and RNA-Seq data for *MCM5* are given as in Figure 3. (C) A schematic shows the Northern blot target locus and primer positions within the ORF. Mitotic (*MCM5*) and meiotic (*mMCM5*) isoforms are given. *ACT1* was used as a loading control. (D) 5′-RACE PCR data are shown for *MCM5* isoforms in rich media (YPD, YPA) and three samples covering the induction of the meiotic isoform (SPII 2, 6, 8 h). (E) A western blot is shown for myc-tagged Mcm5 (Mcm5^myc^) from samples as in panel C except that the 12-h time point in SPII was replaced by a 24-h time point. Pgk1 was used as a loading control. A histogram is shown as in Figure 3D.

In addition, we sought to study a transcript isoform associated with an URS1 motif but showing a middle induction pattern. Therefore, we investigated the conserved cell-cycle regulated gene *MCM5* (initiation of DNA replication), for which we detect an extended 5′-mUTR at the onset of meiotic M-phase (peaking at 6–8 h), but not during starvation or mitotic growth (Figure 5A and B) (see also (26)). To distinguish mitotic and meiotic transcript isoform levels, we performed a Northern blot analysis using RNA samples from cells cultured in growth media (YPD, YPA) and sporulation medium (SPII). Using a probe directed against the internal region of *MCM5* we monitor strong signals corresponding to the mitotic isoform of *MCM5*'s mRNA (*MCM5*) in semi-synchronized cells during pre-meiotic DNA replication (Figure 5C; SPII 2, 4 and 6 h). Once cells exit meiotic M-phase (SPII 8 h onwards), they switch to the longer meiotic isoform (*mMCM5*). The weak signals of both transcript isoforms in mitotic and late meiotic samples may be due to promoter leakage or small populations of cells that either undergo premature sporulation in growth medium or that fail to sporulate in SPII. The middle meiotic induction of a long isoform was confirmed by a 5′-RACE experiment covering the onset of pre-meiotic DNA replication and meiotic M-phase (Figure 5D). The reproducibly low signal for *MCM5* mRNA in cells asynchronously growing in YPD and YPA is due to fluctuating transcript levels during the cell cycle (see Figure 5A). We note that the relative band intensities in the meiotic samples are likely due to slower meiotic kinetics of JHY222. To compare *MCM5* RNA and protein expression profiles, we determined the Mcm5 protein levels in triplicate samples from dividing and differentiating cells using a diploid strain harbouring an allele of *MCM5* with a C-terminal myc tag. The strain displayed normal growth and sporulation properties. As expected, we found peak levels of Mcm5 in rapidly dividing cells cultured in rich media (Figure 5E; YPD, YPA) and during pre-meiotic DNA replication (SPII 4 h). As cells transit through meiotic M-phase and spore formation Mcm5 protein levels decline (SPII6-24). Residual levels of Mcm5 detected in mature asci (SPII 24 h) may stem from a small population of non-sporulating cells in the culture; it is also possible that spores retain some Mcm5 for the first round of DNA replication following germination.

**Figure 6.**
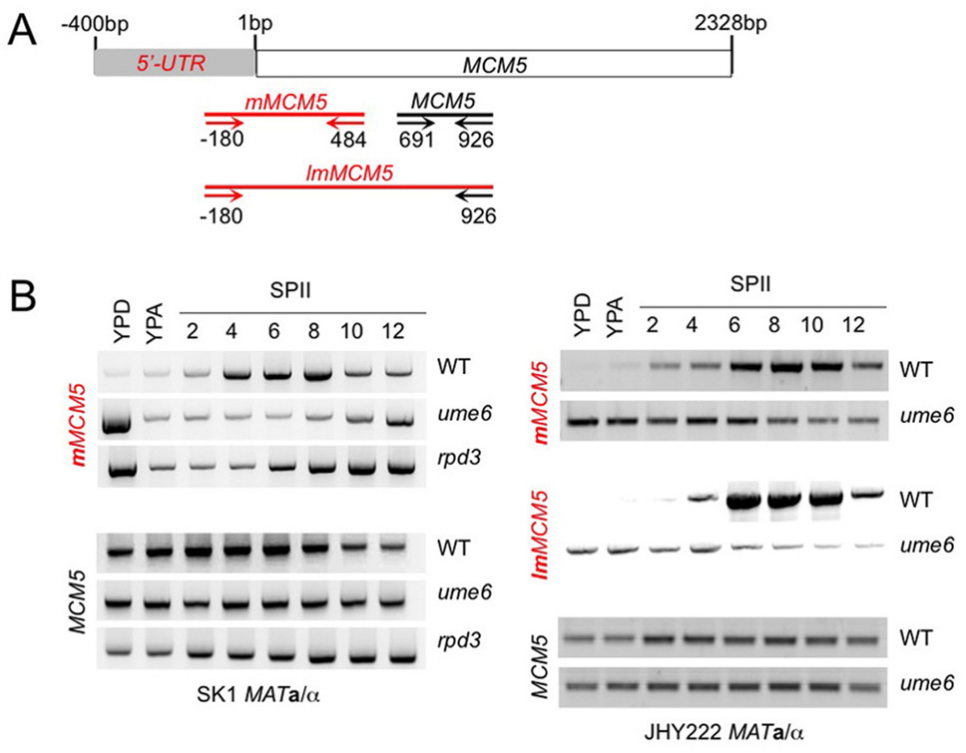
Middle meiotic isoform expression in *rpd3* and *ume6* mutant strains. (A) A schematic shows the *MCM5* locus and primer positions like in Figure 4A. To assay the middle meiotic isoform we used two primer combinations that reveal mUTRs and cover short (*mMCM5*) or long (*lmMCM5*) ORF regions as indicated. (B) The output of RT-PCR assays is shown like in Figure 4B.

The presence of predicted URS1 motifs in the *MCM5* 5′-region—albeit identified only at lower stringency than in the cases of *CFT2* and *RTT10* (Supplementary Additional file 1)—is consistent with earlier work showing that Ume6 controls some middle meiotic promoters (8). Therefore we asked if Ume6 and Rpd3 repressed the *MCM5* 5′-mUTR during mitosis. First, we further validated our high-throughput RNA profiling data, by performing RT-PCR assays using three combinations of primers in SK1 and JHY222 WT strains. Like in the preceding cases, one pair of primers detects the mitotic transcript (*MCM5*) and two other pairs detect the meiotic isoform covering fragments of different lengths (*mMCM5* assayed in SK1; *mMCM5* and *lmMCM5* assayed in JHY222; Figure 6A and B). As expected, the results reproduced the RNA profiling data and the Northern blot (up to a certain extent given that tiling arrays are less sensitive than assays based on PCR or radioactive probes). The slightly delayed onset of 5′-mUTR expression observed in JHY222 reflects its slower sporulation kinetics as compared to SK1 (see Figure 6B *mMCM5* in WT SK1 and *mMCM5/lmMCM5* in WT JHY222).

Then, we compared WT and mutant strains. The RT-PCR data depicted in Figure 6B show that the meiotic isoform is indeed de-repressed in growing SK1 *ume6* and *rpd3* mutants, and in JHY222 *ume6* cells. As expected, homogenous signals were obtained in all samples from WT and mutant strains when the primer pair was located within the ORF (Figure 6B, *MCM5*). It is unclear why the 5′-mUTR signal is much stronger in fermenting cells (YPD) than in respiring cells (YPA), given that primers specific for the *MCM5* ORF yield homogenous transcript levels (Figure 6B). Perhaps the meiotic *MCM5* isoform is somehow stabilized in fermenting *MAT***a**/α *ume6* and *rpd3* mutants.

### URS1 is required for meiotic isoform derepression in *ume6* mutants

**Figure 7.**
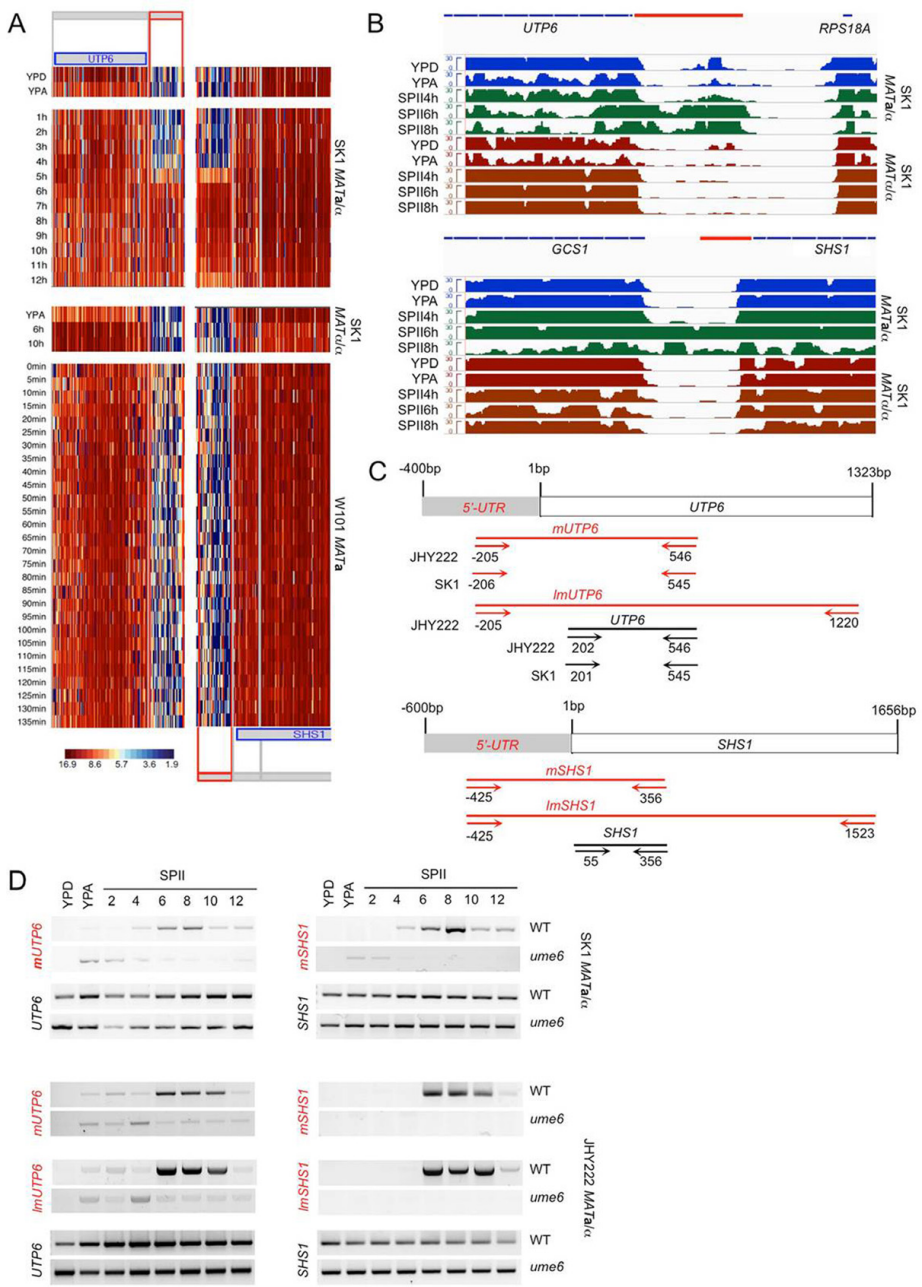
*UTP6* and *SHS1* express middle meiosis-specific isoforms not regulated by Ume6. (A) Heatmaps are shown for 5′-regions of *UTP6* and *SHS1* that lack predicted URS1 motifs. Samples and strains are indicated and the log2 scale is given. (B) RNA-Seq data are shown as in Figure 3B. (C) A schematic shows 5′-UTRs, ORFs and primer positions as in Figure 4A. (D) The output of RT-PCR assays for SK1 and JHY222 WT and mutant (*ume6*) strains is shown for mitotic and meiotic isoforms as in Figure 4B.

We next determined if negative control genes, such as *UTP6* (rRNA processing) and *SHS1* (cytokinesis) lacking predicted URS1 motifs in their promoters (Supplementary Additional file 1), fail to express meiotic isoforms in vegetatively growing *ume6* mutant strains. We first confirmed by RT-PCR in WT SK1 and JHY222 strains the timing of the meiotic isoform's induction and constitutive mRNA expression found with tiling arrays and RNA-Seq (Figure 7A and B). We used two sets of primer pairs detecting the mitotic mRNA (*UTP6*, *SHS1*) and the middle meiotic transcript isoforms (*mUTP6*, *mSHS1*). Here again, we employed two reverse primers located at the extreme 3′-ends of the target transcripts to validate our assumption that the meiotic isoforms span the entire locus (*lmUTP6*, *lmSHS1*) (Figure 7C and D). As expected, we found that neither of the control isoforms containing extended 5′-mUTRs was de-repressed in vegetatively growing cells to a level comparable with *CFT2*, *RTT10* and *MCM5* in SK1 and JHY222 *ume6* mutant cells (Figure 7D). Consistently, the mitotic isoform was expressed in all samples assayed in both strains (Figure 7D). These data show that 5′-mUTRs associated with predicted URS1 motifs are strongly de-repressed in mitosis in the absence of Ume6 and Rpd3, while control transcripts encoded by genes lacking URS1 sites in their promoters are either barely or not detectable in diploid *ume6* mutant cells cultured in media for fermentation, respiration or sporulation.

### Ume6 binds *in vivo* to predicted URS1 motifs in the promoters of *CFT2* and *RTT10*

**Figure 8.**
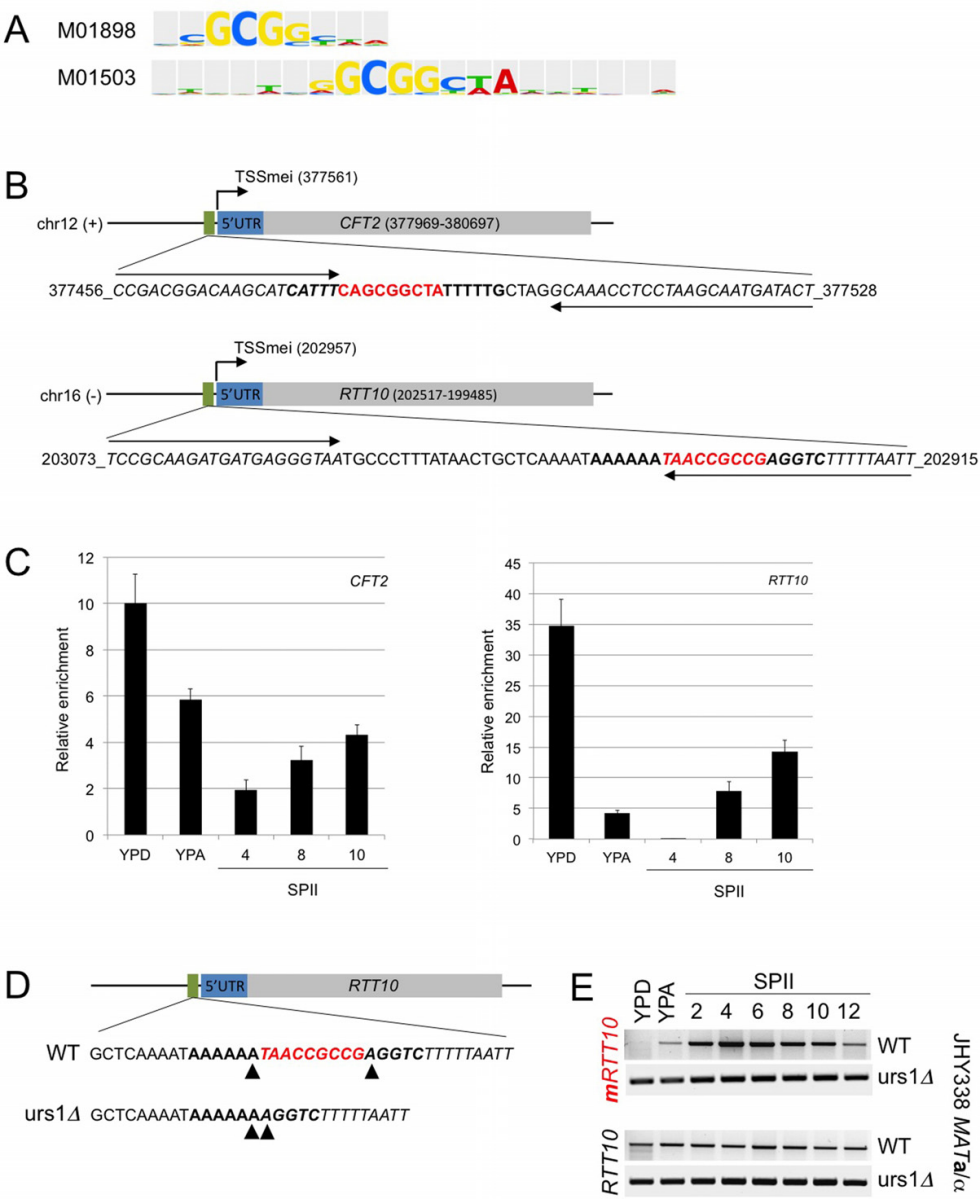
Ume6 *in vivo* binding to predicted URS1 sites and URS1 deletion in the *RTT10* promoter. (A) The logos of two PWMs for the URS1 motif provided by TRANSFAC are shown. The PWM identifier is given to the left. (B) Schematics of the regions containing the predicted URS1s (green rectangle), the UTRs (blue) and the ORFs (grey) are shown. Black lines represent DNA. Chromosome numbers and DNA strands (+, −) are given, and the genome coordinates of the meiotic TSS (TSSmei), the target sequence used for ChIP and the ORF segments are shown. Matches to the complete PWM are given in bold, core URS1 sequences are shown in red. Bases covered by the Q-PCR primers are given in italics. The primer locations are indicated by arrows. (C) Bar diagrams are given of fold-enrichment (*y*-axis) for the *CFT2* and *RTT10* promoters relative to the *NUP85* coding region versus samples from three biological replicates cultured in rich media (YPD, YPA) and sporulation medium (SPII, 4, 8 and 10 h; *x*-axis). The error bars represent the standard deviation. (D) A schematic drawing represents the *RTT10* ORF (grey bar), its 5′-UTR (blue) and the precise DNA sequences of strains bearing the WT sequence and the motif deletion (*urs1Δ*). Arrowheads mark the bases that border the URS1 motif. (E) RT-PCR data showing target gene expression using primers that probe the ORF (*RRT10*) or the 5′-mUTR (*mRRT10*) in samples prepared from diploid strains bearing the WT or the mutated promoter (urs1*Δ*) as indicated. Samples are as in Figure 3. The experiment was done in strains derived from JHY336 and JHY337 that bear the *URA3* auxotrophic marker (see Supplemental Additional Table 1).

Given the expression pattern of early 5′-mUTRs and the mitotic de-repression of meiotic mRNA isoforms in *ume6* and *rpd3* mutants, we determined if Ume6 specifically interacted *in vivo* with the classical URS1 element 5′-TAGCCGCCGA-3′ (9/10 match for *RTT10*) (56), and TRANSFAC URS1 motif M01503 (7/7 match for *CFT2*) (34) (Figure 8A and B). We assayed Ume6-DNA interactions at the *CFT2* and *RTT10* promoters by ChIP and found fluctuating Ume6 *in vivo* binding activities in respiring cells (YPD) as compared to fermenting cells (YPA), and sporulating cells at different stages of meiotic development (SPII; Figure 8C). This pattern corresponds to the known progressive degradation and re-accumulation of Ume6 during the switch from fermentation to respiration and early, middle and late stages of meiosis (12,14). The protein-DNA binding data are coherent with the presence of predicted URS1 target motifs, and the expression of meiotic isoforms during vegetative growth in the absence of Ume6 or Rpd3.

### Mutating the URS1 motif in the *RTT10* promoter mimics the effect of deleting *UME6*

It is possible that Rpd3/Ume6 indirectly control meiotic isoform stability during vegetative growth. If that was the case, mutating the Ume6 target motif should fail to de-repress the meiotic isoform in mitosis. To address this question, we prevented the formation of an active Ume6/Rpd3 repressor complex in the promoter region of *RTT10* by deleting the entire URS1 motif (*urs1Δ*), which fully de-repressed the meiotic isoform in diploid fermenting and respiring cells (Figure 8D and E). This shows, as expected, that deleting *UME6* or the target motif URS1*^RTT10^* bound by Ume6 have the same effect in leading to strong de-repression of the meiotic *RTT10* isoform during vegetative growth.

### Ume6 controls the *in vivo* interaction of RNA polymerase II with developmentally regulated TSSs

**Figure 9.**
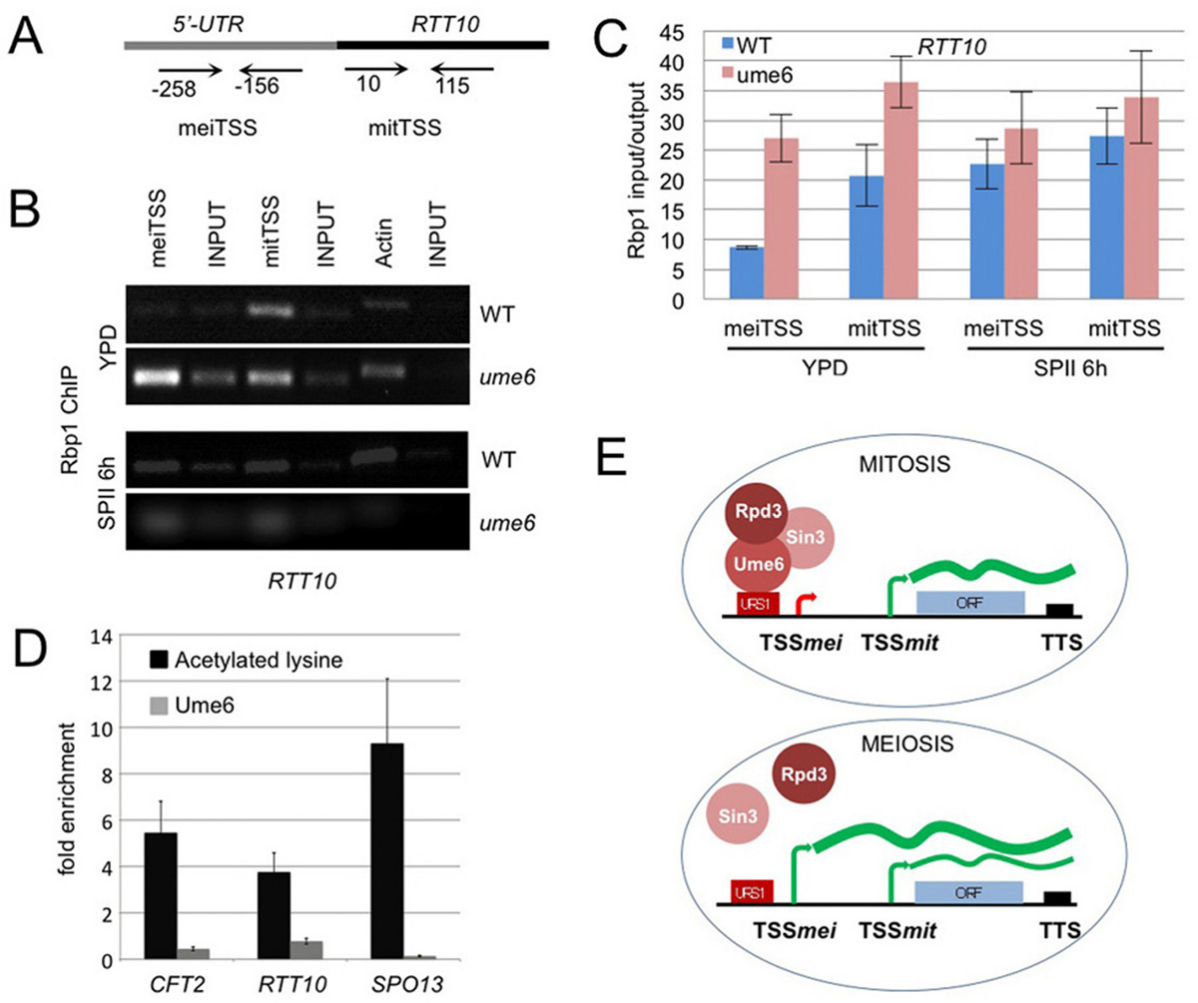
RNA Polymerase II subunit Rpo21/Rbp1 *in vivo* binding and Rpd3 HDAC activity. (A) A schematic shows the locus and the primer positions for mitotic and meiotic TSSs as indicated. (B) RT-PCR-amplified DNA is shown from a ChIP assay revealing RNA polymerase II binding to the mitotic TSS (mitTSS), the meiotic TSS (meiTSS) versus total chromatin (INPUT). Actin was used as a control. Samples were prepared from WT and Ume6 mutant strains (*ume6*) cultured in rich medium (YPD) or sporulation medium for 6 h (SPII 6 h). (C) A colour-coded bar diagram shows the input/output ratio (*y*-axis) versus the target TSSs in the media bound in WT (blue) and mutant (red) strains as detected by Q-PCR. The standard deviation is given. (D) Black bar diagrams show the fold-increase (*y*-axis) of lysine acetylation in an *rpd3* mutant as compared to a WT strain for three target genes (*x*-axis). The standard deviation of two independent biological replicates is given. Light grey bars represent Ume6 binding. The *CFT2* and *RTT10* target genes were compared to the *SPO13* locus, which is repressed in mitosis by Ume6/Rpd3/Sin3. The primers used for *CFT2* and *RTT10* were the same as in Figure 8C, the primers used for *SPO13* were described in (51). (E) The drawing depicts the tripartite repressor complex during mitosis (top) and in early meiosis (bottom) where the DNA binding subunit Ume6 is temporarily degraded by the APC/C. TSSs active during mitosis (TSSmit) or meiosis (TSSmei) are symbolized by arrows. The transcription termination site (TTS) is represented by a black rectangle. A red rectangle indicates the URS1 regulatory motif. A black line is DNA, ORFs are represented by blue rectangles. mRNAs at different concentrations are shown as bold and thin green lines.

We next assayed the *in vivo* binding pattern of RNA polymerase II to the *RTT10* promoter region during rapid mitotic growth in WT cells and a *ume6* mutant strain, using an antibody against its major subunit Rpo21/Rbp1. We find that RNA polymerase II preferentially interacts with the mitotic TSS in vegetatively growing WT cells and the meiotic TSS in sporulating cells, while it strongly binds to both meiotic and mitotic TSSs in dividing *ume6* mutant cells (Figure 9A–C). The data are thus consistent with a model positing a Ume6/Rpd3-dependent epigenetic repressor mechanism for meiotic isoforms during mitotic growth.

### Rpd3 acts as a deacetylase *in vivo* on the *CFT2* and *RTT10* promoters

Finally, we sought to confirm that Rpd3 indeed exerts its known HDAC activity on the target promoters in WT versus *rpd3* mutant cells (Figure 9D). Using an antibody against acetylated lysine, we find that *CFT2* and *RTT10* promoters display substantially increased levels of protein acetylation at the predicted target URS1 motifs in the absence of Rpd3/Ume6 during mitotic growth (Figure 9D, see also Figure 8A–C). Previous work has shown that the early meiotic gene *SPO13* is a direct target of Ume6-directed Rpd3 deacetylation, we therefore used this gene as a positive control (51).

Taken together, our results are consistent with the notion that the HDAC Rpd3, its DNA binding subunit Ume6 and by inference Sin3, directly repress certain early and middle meiotic transcript isoforms during vegetative growth by interacting with URS1 motifs. After the onset of meiosis, the APC/C degrades Ume6, and the HDAC complex can no longer repress the meiotic isoform (14), which is then co-expressed with the mitotic transcript or may even become the predominant mRNA encoded by the locus. This epigenetic mechanism enables cells to express meiotic transcript isoforms containing extended 5′-mUTRs as they progress through early and middle stages of the meiotic developmental pathway (Figure 9E).

## DISCUSSION

This study addresses the question of what mechanism controls the dynamic expression of mitotic and meiotic transcript isoforms during vegetative growth and gametogenesis in a simple eukaryote. To this end, we have analysed a comprehensive tiling array data set which includes samples from fermenting haploid and diploid strains, as well as respiring, starving and sporulating diploid cell populations (28). We found that extended 5′- and 3′-mUTRs of constitutively expressed transcripts show early, middle and late induction patterns that correspond to those reported for meiotically up-regulated mRNAs (3). Validation experiments carried out in two different strain backgrounds (SK1 and JHY222 derived from the reference strain S288c; (28)) confirmed our assumption that temporary 5′-extensions reflect the expression of distinct stage-specific transcript isoforms in three cases. We determined that predicted URS1 motifs are statistically significantly enriched within the up-stream regions of candidate genes, and discovered a novel role for the conserved Rpd3/Sin3/Ume6 regulatory complex in the mitotic repression of early meiotic transcript isoforms.

It has been known for many years that epigenetic modifications, such as histone deacetylation mediated by Rpd3 and its DNA-binding partner Ume6, are essential for the full repression of meiosis-specific genes during mitotic growth (6,7,57,58). However, studies aimed at identifying genes directly regulated by Rpd3 and Ume6 yielded only partially overlapping sets of target loci, indicating that Ume6 bound promoters of genes that were not de-repressed in its absence (and vice versa) (9,59,60). The data reported here offer a plausible explanation for why many promoters containing URS1 motifs bound by Ume6 *in vivo* are not de-repressed in *ume6* mutant cells profiled with Ye6100 GeneChips and Yeast 2.0 arrays (8–10): these microarrays reveal differentially expressed genes, but they are unable to detect complex changes in transcript architecture especially at the 5′-end, because the target sequences covered by their probes are typically located within 3′-regions of mRNAs (see Supplementary Additional file 4A and B; the full data set will be published elsewhere; Lardenois *et al.*, in preparation).

While expression data in WT and mutant cells, motif predictions and Ume6 binding activities are coherent for *CFT2* and *RTT10*, the case of *MCM5* is more complicated. We identified a URS1 motif in the *MCM5* up-stream region, albeit at a less stringent level than in the case of the other two loci, and the meiotic isoform is de-repressed strongly in fermenting *ume6* and *rdp3* cells. However, the meiotic isoform of *MCM5* is induced during M-phase, and therefore corresponds to the middle meiotic pattern of which Ume6 is not the predominant regulator (8). Furthermore, Ume6 binds to the motif *in vivo* only very weakly (M. Law, unpublished observation). It is therefore conceivable that the developmentally controlled isoform of *MCM5* is regulated by additional factors that influence the expression of middle meiotic isoforms.

We observe for *UTP6* and *SHS1*, two loci that lack predicted URS1 motifs upstream of their middle 5′-mUTRs, only extremely weak or no expression of the meiotic isoform during fermentation, respiration and sporulation. Contrary to early isoforms, which are de-repressed in *ume6* mutant cells cultured in rich medium and sporulation medium, it appears that the developmental arrest phenotype of *ume6* mutant cells prevents the transcriptional induction of extended *UTP6* and *SHS1* transcripts (58). It will be interesting to determine which regulators are involved in the mitotic repression and meiotic activation of middle meiotic isoforms.

We propose that the conserved HDAC/repressor complex Rpd3/Sin3/Ume6 has two complementary roles relevant for gametogenesis that both involve Rpd3's histone deacetylation activity: the first is to repress entire transcripts encoded by meiosis-specific genes during mitosis, and the second is to prevent expression of meiotic transcript isoforms possessing extended 5′-mUTRs in vegetatively growing cells, by rendering their cryptic TSSs inaccessible via interaction with URS1 motifs. This negative regulation is then relieved during early meiosis (Figure 9E).

The data imply that the expression of stage-specific transcript isoforms is part of the overall meiotic expression program. In principle, such a phenomenon should not be limited to early meiosis and Ume6-dependent 5′-mUTRs, because the Rpd3 core complex directly interacts with multiple DNA binding proteins (61). It is therefore possible that Rpd3 is also relevant for the regulation of middle and perhaps late 5′-mUTRs. In addition, the protein might be involved in a UTR-mediated response to stress, starvation or developmental pathways, such as filamentous growth that require complex gene expression programs (62,63).

Why do certain genes encode transcripts that change in length when cells exit mitosis and enter gametogenesis? The expression of meiotic isoforms might provide the cells with a mechanism to ensure continued gene expression during cell differentiation when the mitotic TSS is somehow inactivated. Alternatively, certain 5′-mUTRs (and 3′-mUTRs) could positively or negatively influence protein levels in sporulating yeast cells. Based on large-scale RNA-ribosome association profiling data it was proposed that 5′-leader extensions including so-called AUG uORFs can diminish mRNA translation during meiosis by competing with the main ORF; such a mechanism was predicted to down-regulate protein levels, among others, in the cases of Cft2, Rtt10 and Mcm5 (26). Our western blot data indicate that the former two proteins are stabilized in acetate and further up-regulated during sporulation, before the meiotic isoform accumulates to levels detectable by our assays. Moreover, Rtt10 strongly accumulates in *ume6* cells cultured in YPD. These patterns might arise for several reasons that are not mutually exclusive: Ume6 might be a negative regulator of Rtt10, acetate may somehow increase the half-life of Cft2 and Rtt10, or the meiotic isoforms encoding these proteins are translated extremely efficiently. The protein profiles we observe make sense from a biological point of view since Cft2 is involved in mRNA 3′-end modification and Rtt10 is required for protein recycling, both of which are processes active in gametogenesis. Likewise, Mcm5 levels decline—in this case in a coherent fashion with the predominant expression of the meiotic isoform containing AUG uORFs—as cells exit pre-meiotic DNA replication and transit through the meiotic divisions without an intervening S-phase. We note that RNA-ribosome association appears not to be an optimal measure for protein translation as opposed to RNA-ribosome dissociation at genuine stop codons (64). More work, including large-scale protein profiling of mitosis versus meiosis (65) and individual analyses of target genes, will be required to elucidate the various roles that extended 5′-UTRs may play during meiosis and gametogenesis.

The information presented in this study has two major corollaries. First, the transcriptome of gametogenesis comprises specifically induced meiotic transcript isoforms (19,26), which show early, middle and late induction patterns like mRNAs (4,5), and ncRNAs (28). Secondly, the conserved HDAC Rdp3 and its DNA binding subunit Ume6 play a novel role in repressing early meiotic transcript isoforms during vegetative growth via en epigenetic transcriptional mechanism that involves direct interaction with the URS1 motif, which controls access of RNA polymerase II to its target TSSs.

Recent analyses in fly, zebrafish and mammalian organisms have revealed that eukaryotic promoters typically contain multiple TSSs yielding highly variable numbers of transcript isoforms encoded in the genomes during growth and development (66–68). UTRs fulfil regulatory functions during mammalian processes, such as spermatogenesis (69,70), and a recent study has proposed differential TSS selection to be important for the control of gene expression in the mouse male germline (71). Moreover, HDAC/Sin3 genes are transcribed in the male gonad (72–74), and in non-testicular somatic tissues where they are associated with cancer (75–79). Our findings therefore have implications for the regulatory mechanism underlying dynamic transcript architecture important for development and disease in higher eukaryotes.

## SUPPLEMENTARY DATA

Supplementary Data are available at NAR Online.

SUPPLEMENTARY DATA
