# Supplementary material for: The conserved histone deacetylase Rpd3 and its DNA binding subunit Ume6 control dynamic transcript architecture during mitotic growth and meiotic development
Source: Nucleic Acids Res. 2014 Dec 3;43(1):115–28. doi: 10.1093/nar/gku1185 (PMC4288150; doi:10.1093/nar/gku1185)
Supplement: SUPPLEMENTARY DATA [file supp_43_1_115__index.html]

The conserved histone deacetylase Rpd3 and its DNA binding subunit Ume6 control dynamic transcript architecture during mitotic growth and meiotic development — The conserved histone deacetylase Rpd3 and its DNA binding subunit Ume6 control dynamic transcript architecture during mitotic growth and meiotic development — The conserved histone deacetylase Rpd3 and its DNA binding subunit Ume6 control dynamic transcript architecture during mitotic growth and meiotic development — SUPPLEMENTARY DATA 

# The conserved histone deacetylase Rpd3 and its DNA binding subunit Ume6 control dynamic transcript architecture during mitotic growth and meiotic development

## SUPPLEMENTARY DATA

**Files in this Data Supplement:**

- SUPPLEMENTARY DATA
- SUPPLEMENTARY DATA
- SUPPLEMENTARY DATA
- SUPPLEMENTARY DATA
- SUPPLEMENTARY DATA
- SUPPLEMENTARY DATA
- SUPPLEMENTARY DATA
- SUPPLEMENTARY DATA
- SUPPLEMENTARY DATA
- SUPPLEMENTARY DATA
- SUPPLEMENTARY DATA
- SUPPLEMENTARY DATA
- SUPPLEMENTARY DATA
