# Supplementary material for: The conserved histone deacetylase Rpd3 and its DNA binding subunit Ume6 control dynamic transcript architecture during mitotic growth and meiotic development
Source: Nucleic Acids Res. 2014 Dec 3;43(1):115–28. doi: 10.1093/nar/gku1185 (PMC4288150; doi:10.1093/nar/gku1185)
Supplement: SUPPLEMENTARY DATA [file supp_gku1185_Additional-Table-1mp.doc]

| Strain ID | Background and genotype | Reference |
| --- | --- | --- |
| MPY1 | JHY222 *MAT***a**/*MAT*α *HAP1*/*HAP1 MKT1*(D30G)/*MKT1*(D30G) *RME1*(INS 308A)/*RME1*(INS 308A) *TAO3*(E1493Q)/*TAO3*(E1493Q) | (1) |
| MPY70 | SK1 *MAT***a**/*MATα ho::LYS2*/*ho::LYS2 ura3*/*ura3 lys2*/*lys2 leu2::hisG*/*leu2::hisG arg4-Nsp*/*arg4-Bgl his4x::LEU2-URA3*/*his4B::LEU2* | (2) |
| MPY441 | SK1 *MAT****a***/*MATα ho::hisG*/*ho::hisG lys2*/*lys2 ura3*/*ura3 leu2::hisG*/*leu2::hisG arg4-Nsp*/*arg4-Bgl his4x::LEU2-URA3*/*his4B::LEU2 trp1::hisG*/*trp1::hisG rpd3::KanMX4*/*rpd3::KanMX4* | (3) |
| MPY542 | *MAT***a**/*MAT*α *HAP1*/*HAP1 MKT1*(D30G)/*MKT1*(D30G) *RME1*(INS 308A)/*RME1*(INS 308A) *TAO3*(E1493Q)/*TAO3*(E1493Q) *ume6::KanMX4/ume6::KanMX4* | This study |
| MPY631 | *JHY222 MAT****a****/MATα HAP1/HAP1 MKT1(D30G)/MKT1(D30G) RME1 (INS 308A)/ RME1(INS 308A) TAO3 (E1493Q)/ TAO3 (E1493Q) sum1::KanMX4/ sum1::KanMX4* | (1) |
| MPY702 | SK1 *MAT****a***/*MATα ura3*/*ura3 leu2*/*leu2 trp1*/*trp1 lys2*/*lys2 ho::LYS2*/*ho::LYS2 gal80::LEU2*/*gal80::LEU2 ume6::TRP1*/*ume6::TRP1* | (4) |
| MPY732 | SK1 *MAT***a**/*MAT*α *ho::LYS2*/*ho::LYS2* *ura3*/*ura3* *lys2*/*lys2* *leu2::hisG*/*leu2::hisG* *arg4-Nsp*/*arg4-Bgl* *his4x::LEU2-URA3*/*his4B::LEU2* *sum1*::KanMX4/*sum1*::kanMX4 | (1) |
| MPY740 | JHY336 *MAT***a** *ura3 leu2 his3* | (5) |
| MPY741 | JHY337 *MAT*α *ura3 leu2 lys2* | (5) |
| MPY742 | JHY338 *MAT***a**/*MAT*α *ura3/ura3 leu2/leu2 lys2/LYS2 his3/HIS3* | (5) |
| MPY749 | *MAT***a** *ura3 leu2 his3* urs1Δ*^RTT10^* | This study |
| MPY751 | *MAT*α *ura3 leu2 lys2* urs1Δ*^RTT10^* | This study |
| MPY753 | *MAT***a**/*MAT*α *ura3/ura3 leu2/leu2 lys2/LYS2 his3/HIS3* urs1Δ*^RTT10^*/urs1Δ*^RTT10^* | This study |
| MPY762 | *MAT***a**/*MAT*α *HAP1*/*HAP1 MKT1*(D30G)/*MKT1*(D30G) *RME1*(INS 308A)/*RME1*(INS 308A) *TAO3*(E1493Q)/*TAO3*(E1493Q) *RTT10*/*RTT10*-Myc::KanMX4 | This study |
| MPY763 | *MAT***a**/*MAT*α *HAP1*/*HAP1 MKT1*(D30G)/*MKT1*(D30G) *RME1*(INS 308A)/*RME1*(INS 308A) *TAO3*(E1493Q)/*TAO3*(E1493Q) *CFT2*/*CFT2*-Myc::KanMX4 | This study |
| MPY764 | *MAT***a**/*MAT*α *HAP1*/*HAP1 MKT1*(D30G)/*MKT1*(D30G) *RME1*(INS 308A)/*RME1*(INS 308A) *TAO3*(E1493Q)/*TAO3*(E1493Q) *MCM5*/*MCM5*-Myc::KanMX4 | This study |
| MPY768 | *MAT***a**/*MATα ho::LYS2*/*ho::LYS2 ura3*/*ura3 lys2*/*lys2 leu2::hisG*/*leu2::hisG arg4-Nsp*/*arg4-Bgl his4x::LEU2-URA3*/*his4B::LEU2 RTT10*/*RTT10*-Myc::KanMX4 | This study |
| MPY769 | *MAT****a***/*MATα ura3*/*ura3 leu2*/*leu2 trp1*/*trp1 lys2*/*lys2 ho::LYS2*/*ho::LYS2 gal80::LEU2*/*gal80::LEU2 ume6::TRP1*/*ume6::TRP1 RTT10*/*RTT10*-Myc::KanMX4 | This study |

1. Lardenois, A., Liu, Y., Walther, T., Chalmel, F., Evrard, B., Granovskaia, M., Chu, A., Davis, R.W., Steinmetz, L.M. and Primig, M. (2011) Execution of the meiotic noncoding RNA expression program and the onset of gametogenesis in yeast require the conserved exosome subunit Rrp6. *Proc Natl Acad Sci U S A*, **108**, 1058-1063.

2. Primig, M., Williams, R.M., Winzeler, E.A., Tevzadze, G.G., Conway, A.R., Hwang, S.Y., Davis, R.W. and Esposito, R.E. (2000) The core meiotic transcriptome in budding yeasts. *Nat Genet*, **26**, 415-423.

3. Burgess, S.M., Ajimura, M. and Kleckner, N. (1999) GCN5-dependent histone H3 acetylation and RPD3-dependent histone H4 deacetylation have distinct, opposing effects on IME2 transcription, during meiosis and during vegetative growth, in budding yeast. *Proceedings of the National Academy of Sciences of the United States of America*, **96**, 6835-6840.

4. Shimizu, M., Takahashi, K., Lamb, T.M., Shindo, H. and Mitchell, A.P. (2003) Yeast Ume6p repressor permits activator binding but restricts TBP binding at the HOP1 promoter. *Nucleic Acids Res*, **31**, 3033-3037.

5. Horecka, J. and Davis, R. (2013) The 50:50 method for PCR-based seamless genome editing in yeast. *Yeast*.
