# Supplementary material for: The conserved histone deacetylase Rpd3 and its DNA binding subunit Ume6 control dynamic transcript architecture during mitotic growth and meiotic development
Source: Nucleic Acids Res. 2014 Dec 3;43(1):115–28. doi: 10.1093/nar/gku1185 (PMC4288150; doi:10.1093/nar/gku1185)
Supplement: SUPPLEMENTARY DATA [file supp_gku1185_Additional-Table-2.doc]

| **ORF** | **Cassette**  **plasmid/**  **template** | **C-**  **terminal**  **tag** | **Forward primer** | **Reverse primer** |
| --- | --- | --- | --- | --- |
| *CFT2* | pYM18 | Myc | 5’-ACTTTTTGATACTGTCAAAAAATTGGTTACGGATATGTTAGCAAAAATCCGTACGCTGCAGGTCGAC-3’ | 5’-TCCACGAACGTAAATTGAACCTTTTATTTGTGCTGTATACAAGAAGGCTAATCGATGAATTCGAGCTCG-3’ |
| *RTT10* | pYM18 | Myc | 5’-CTGAGAAAACGTTACTGATCGGAGGTGTTGGTTTATCAATTTGGAAAAAACGTACGCTGCAGGTCGAC-3’ | 5’-TAATACATATCTATAATATTCTTTTATGTATATGTTAATTTTTTGTATCAATCGATGAATTCGAGCTCG-3’ |
| *MCM5* | pYM18 | Myc | 5’-AAACAATTCAATTGAGACACCAGGGACAGAATATTTACAGAAGTGGTGTACGTACGCTGCAGGTCGAC-3’ | 5’-TACATGCAAACAAGTAGAAAAGGCGTCAAGCTAAGACTTTATTGTTGTCAATCGATGAATTCGAGCTCG-3’ |
