# Supplementary material for: The conserved histone deacetylase Rpd3 and its DNA binding subunit Ume6 control dynamic transcript architecture during mitotic growth and meiotic development
Source: Nucleic Acids Res. 2014 Dec 3;43(1):115–28. doi: 10.1093/nar/gku1185 (PMC4288150; doi:10.1093/nar/gku1185)
Supplement: SUPPLEMENTARY DATA [file supp_gku1185_Additional-Table-3.doc]

| **Target** | **Forward primer** | **Reverse primer** | **Size (bp)** |
| --- | --- | --- | --- |
| *Meiotic TSS RTT10* | 5’-AGTCGTGCTTTGGTGAGTTG | 5’-CGCCGATGTGTATGCTAGG | 103 |
| *Mitotic TSS RTT10* | 5’-TTGTCTCATTATGGTCCTGCG | 5’-TCAACGTAGCCGAATGATAGTC | 106 |
| *RDN25-1* | 5’-AGTGACGAAGCCTAGACCGT | 5’-AGGATCGACTAACCCACGTC | 148 |
| *ACT1* | 5’-TGGATTCTGGTATGTTCTAGCG | 5’-GGAGGTTATGGGAGAGTGAAAA | 126 |
