# Supplementary material for: The conserved histone deacetylase Rpd3 and its DNA binding subunit Ume6 control dynamic transcript architecture during mitotic growth and meiotic development
Source: Nucleic Acids Res. 2014 Dec 3;43(1):115–28. doi: 10.1093/nar/gku1185 (PMC4288150; doi:10.1093/nar/gku1185)
Supplement: SUPPLEMENTARY DATA [file supp_gku1185_Additional-Table-4.doc]

| **Target** | **Forward primer** | **Reverse primer** | **Size** |
| --- | --- | --- | --- |
| *ACT1* | 5’-CTCGTGCTGTCTTCCCATCT-3’ | 5’-AGATGGACCACTTTCGTCGT-3’ | 1025 bp |
| *MCM5* | 5’-CGCATCAACAGGAGAACAGA-3’ | 5’-TCCGGAGACTTGATGCTCTT-3’ | 434 bp |
