# Supplementary material for: The conserved histone deacetylase Rpd3 and its DNA binding subunit Ume6 control dynamic transcript architecture during mitotic growth and meiotic development
Source: Nucleic Acids Res. 2014 Dec 3;43(1):115–28. doi: 10.1093/nar/gku1185 (PMC4288150; doi:10.1093/nar/gku1185)
Supplement: SUPPLEMENTARY DATA [file supp_gku1185_Additional-Table-5.doc]

| **Name** | **Purpose** | **Reverse primer** |
| --- | --- | --- |
| *CFT2* GSP1 | cDNA synthesis | 5’-AAGCTCGGCTTGGTCAAAAT-3’ |
| *RTT10* GSP1 |  | 5’-CACCGAATGAAAACCATTGA-3’ |
| *MCM5* GSP1 |  | 5’-AGGGGGCAATAGAGTTGGTC-3’ |
| *CFT2* GSP2 | Diagnostic PCR | 5’-TTGAGAGGAGCCAAACCTGT-3’ |
| *RTT10* GSP2 |  | 5’-GGCAACGTTTTCTCGTGATT-3’ |
| *MCM5* GSP2 |  | 5’-AACGGACAATCTTGGAGACG-3’ |
