# Supplementary material for: The conserved histone deacetylase Rpd3 and its DNA binding subunit Ume6 control dynamic transcript architecture during mitotic growth and meiotic development
Source: Nucleic Acids Res. 2014 Dec 3;43(1):115–28. doi: 10.1093/nar/gku1185 (PMC4288150; doi:10.1093/nar/gku1185)
Supplement: SUPPLEMENTARY DATA [file supp_gku1185_Additional-Table-6.doc]

| **Target**  **genes** | **Forward**  **primer** | **Reverse**  **primer** | **Size (bp)** |
| --- | --- | --- | --- |
| *CFT2* | 5’-CCATTGACTCCAACCTCGAT-3’ | 5’-ATTGTTCACTTGCGGTAGGC-3’ | 113 |
| *mCFT2* | 5’-CTTTGCTCCCAAATGCAAGT-3’ | 5’-CCCAGTACTTGATGCATTGCT-3’ | 495 |
| *lmCFT2* | 5’-CTTTGCTCCCAAATGCAAGT-3’ | 5’-ATTGTTCACTTGCGGTAGGC-3’ | 2641 |
| *RTT10* | 5’TTGGTTGACCACAAACTGGA-3’ | 5’-ACCGGACGTTATTGTCGAAG-3’ | 252 |
| *mRTT10* | 5’-TTCACGTAGTCGTGCTTTGG-3’ | 5’-CTTGCACCATAGGCCAAAAT-3’ | 466 |
| *lmRTT10* | 5’-TTCACGTAGTCGTGCTTTGG-3’ | 5’-ACCGGACGTTATTGTCGAAG-3’ | 3091 |
| *MCM5* | 5’-TGGTGATGAATCGACCAAGA-3’ | 5’-GATCCGGCACCATTTTTAGA-3’ | 246 |
| *mMCM5* | 5’-GTTTGTTGCTATGCGGTGTG-3’ | 5’-AACGGACAATCTTGGAGACG-3’ | 665 |
| *lmMCM5* | 5’-GTTTGTTGCTATGCGGTGTG-3’ | 5’-GATCCGGCACCATTTTTAGA-3’ | 1106 |
| *UTP6* | 5’-GCCAAGCGGTGTAAGAGAAT-3’ | 5’-CCATAGCTTAGGCACATCAGG-3’ | 345 |
| *mUTP6* | 5’-TTTTCCTGCGCTGTCAGATA-3’ | 5’-CCATAGCTTAGGCACATCAGG-3’ | 752 |
| *lmUTP6* | 5’-TTTTCCTGCGCTGTCAGATA-3’ | 5’-TTCAATGACTTCACCGATGC-3’ | 1425 |
| *SHS1* | 5’-AAACGTGGGATCACATACACA -3’ | 5’-CCTTGATCTTTGTTGCCCCC-3’ | 302 |
| *mSHS1* | 5’-ATACCGGCACATTCAAGGCA-3’ | 5’-CCTTGATCTTTGTTGCCCCC-3’ | 782 |
| *lmSHS1* | 5’-ATACCGGCACATTCAAGGCA-3’ | 5’-TCCCTCAATTTCAATTCGTGT-3’ | 1948 |
