# Supplementary material for: The conserved histone deacetylase Rpd3 and its DNA binding subunit Ume6 control dynamic transcript architecture during mitotic growth and meiotic development
Source: Nucleic Acids Res. 2014 Dec 3;43(1):115–28. doi: 10.1093/nar/gku1185 (PMC4288150; doi:10.1093/nar/gku1185)
Supplement: SUPPLEMENTARY DATA [file supp_gku1185_Additional-Table-7.doc]

| Target | Forward primer | Reverse primer |
| --- | --- | --- |
| *CFT2* | 5’- CCGACGGACAAGCATCATTT-3’ | 5’-AAGTATCATTGCTTAGGAGGTTTGC-3’ |
| *RTT10* | 5’- TCCGCAAGATGATGAGGGTAA-3’ | 5’-AATTAAAAAGACCTCGGCGGTTA-3’ |
| *NUP85* | 5’-TTCGCGAAGGAGCATAATGC-3’ | 5’-ACACTTCCAATTCATTCAGAATCG-3’ |
