# Supplementary material for: The conserved histone deacetylase Rpd3 and its DNA binding subunit Ume6 control dynamic transcript architecture during mitotic growth and meiotic development
Source: Nucleic Acids Res. 2014 Dec 3;43(1):115–28. doi: 10.1093/nar/gku1185 (PMC4288150; doi:10.1093/nar/gku1185)
Supplement: SUPPLEMENTARY DATA [file supp_gku1185_Additional-Table-8.doc]

| Name | Purpose | Sequence |
| --- | --- | --- |
| RTT10 50/50-U2 | 50/50 forward primer to amplify *URA3* from pJH136 | 5’TCCGCAAGATGATGAGGGTAATGCCCTTTATAACTGCTCAAAATAAAAAAAGGTCTTTTTAATTATCAAGGTTATAAGAAGTGCCAGAAGTGGGTGAACTCGTACGCTGCAGGTCGAC-3’ |
| D2-50 RTT10 | 50/50 reverse primer to amplify *URA3* from pJH136 | 5’GTTCACCCACTTCTGGCACTTCTTATAACCTTGATAATTAAAAAGACCTATCGATGAATTCGAGCTCG-3’ |
| URA3.for | Forward primer to confirm integration of the cassette into the genome | 5’-CACAGTTAAGCCGCTAAAGGC-3’ |
| URA3.rev | Reverse primer to confirm integration of the cassette into the genome | 5’-AGTATATTCTCCAGTAGCTAGGGAGCC-3’ |
| RTT10seq.for | Forward primer to confirm URS1 deletion | 5’-CGTCCTATTTCCGCAAGATG-3’ |
| RTT10seq.rev | Reverse primer to confirm URS1 deletion | 5’-GGCAAGACCAGACTCAGTTTC-3’ |
