# Supplementary figures and images for: The conserved histone deacetylase Rpd3 and its DNA binding subunit Ume6 control dynamic transcript architecture during mitotic growth and meiotic development
Source: Nucleic Acids Res. 2014 Dec 3;43(1):115–28. doi: 10.1093/nar/gku1185 (PMC4288150; doi:10.1093/nar/gku1185)

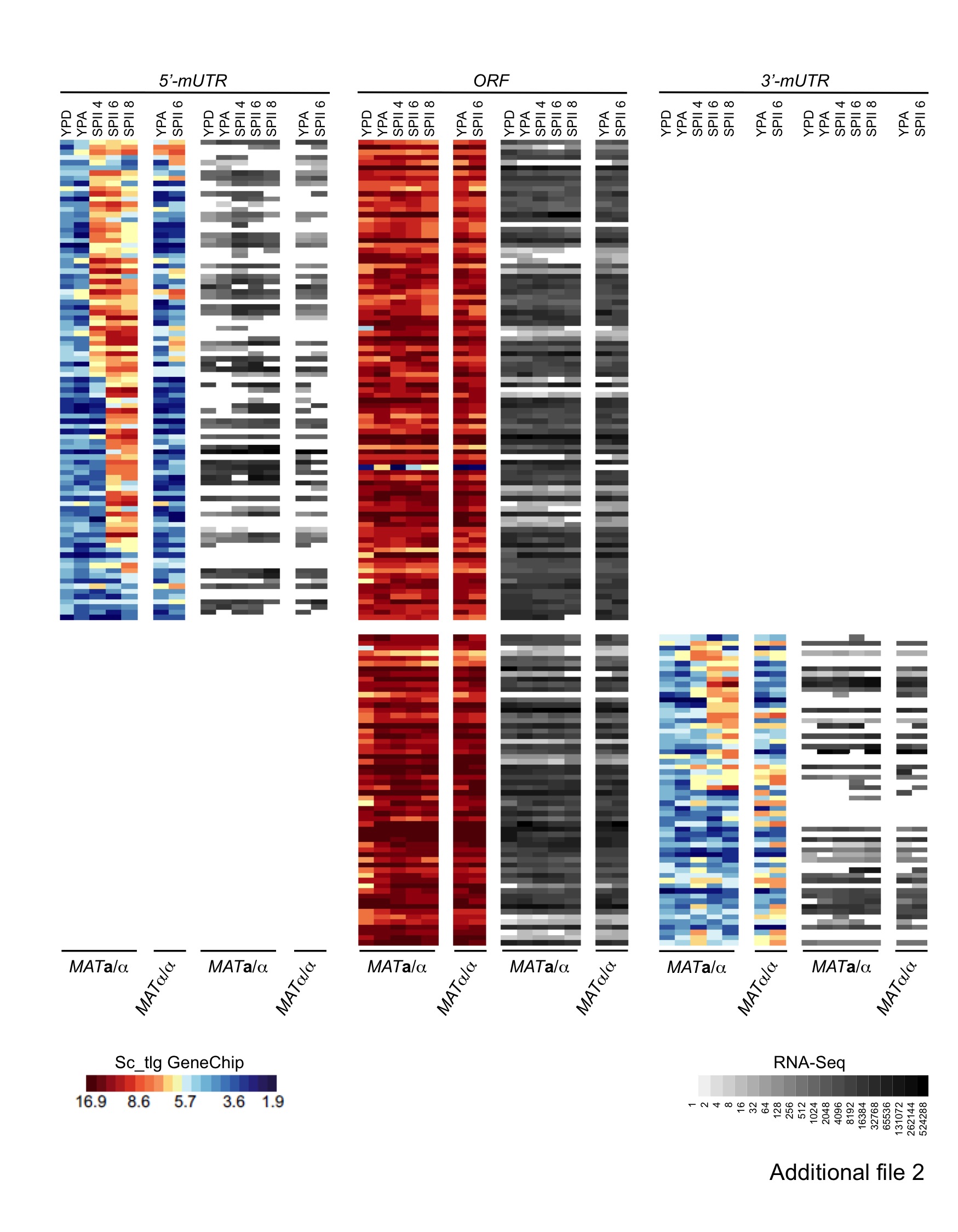

Supplement: SUPPLEMENTARY DATA [file supp_gku1185_nar-00300-x-2014-File012.jpg]

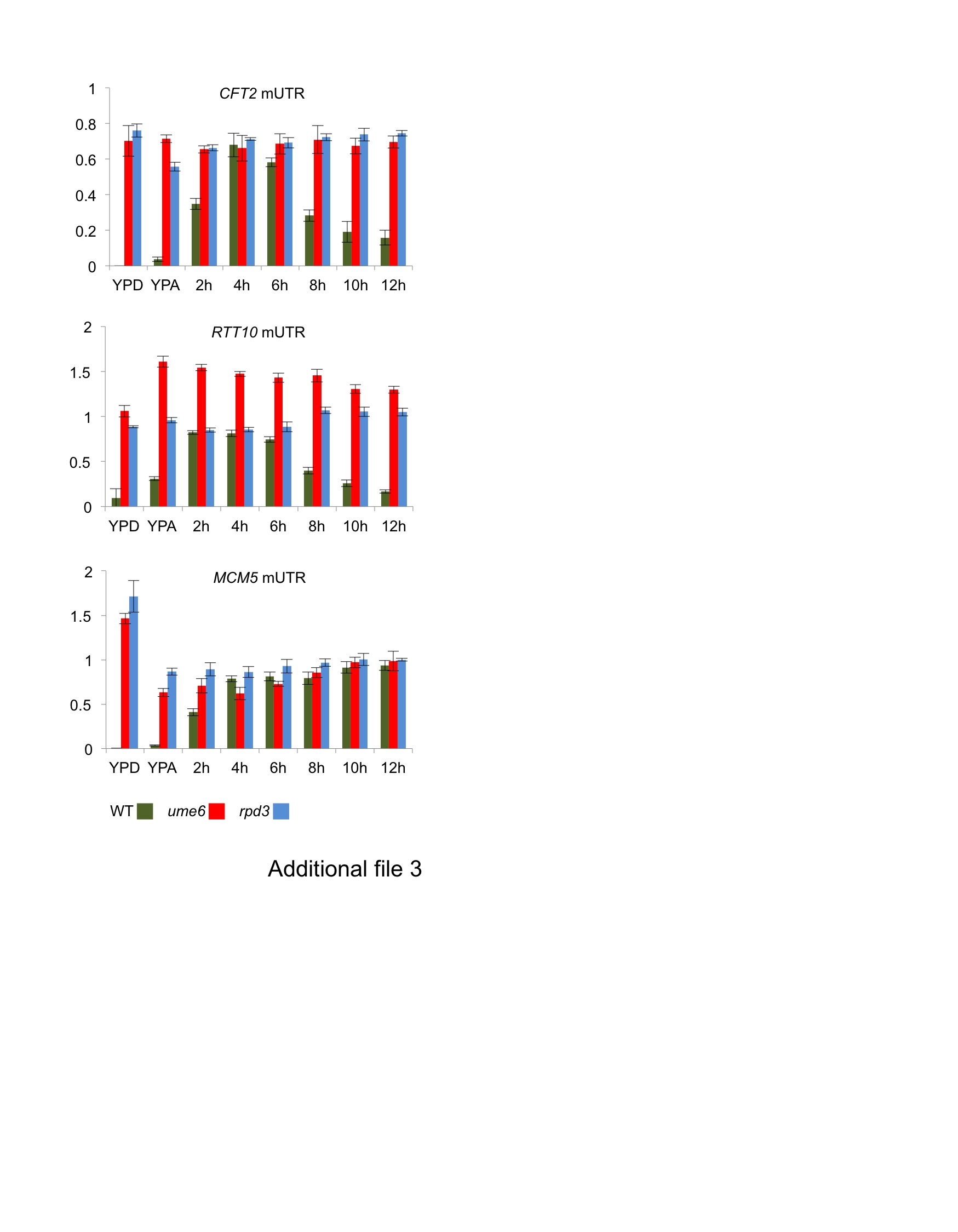

Supplement: SUPPLEMENTARY DATA [file supp_gku1185_nar-00300-x-2014-File013.jpg]

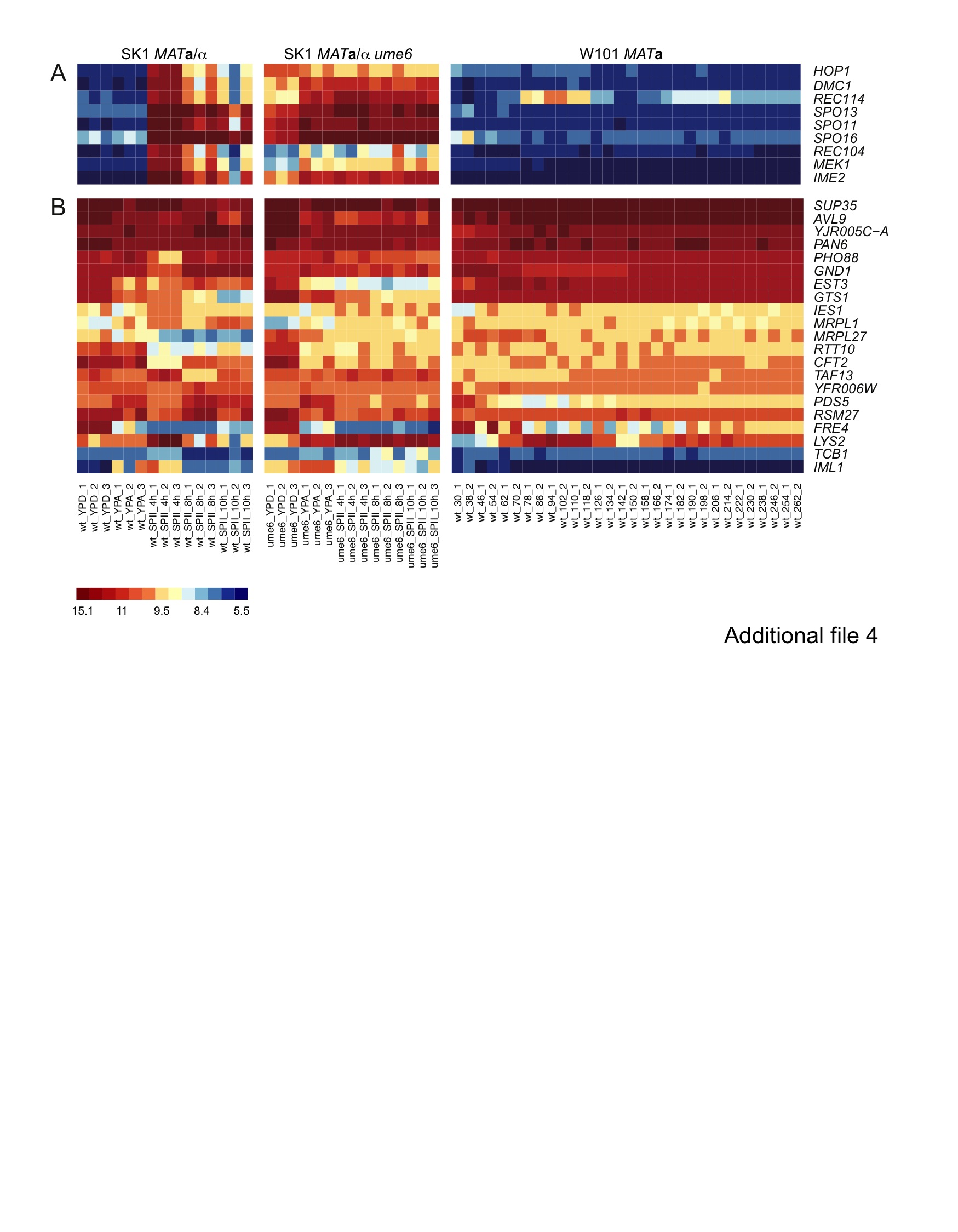

Supplement: SUPPLEMENTARY DATA [file supp_gku1185_nar-00300-x-2014-File014.jpg]
