# Supplementary material for: The conserved histone deacetylase Rpd3 and its DNA binding subunit Ume6 control dynamic transcript architecture during mitotic growth and meiotic development
Source: Nucleic Acids Res. 2014 Dec 3;43(1):115–28. doi: 10.1093/nar/gku1185 (PMC4288150; doi:10.1093/nar/gku1185)
Supplement: SUPPLEMENTARY DATA [file supp_gku1185_nar-00300-x-2014-File023_mp.docx]

ADDITIONAL MATERIAL

Additional file 1. Target gene information. The file contains annotation data for 5’- and 3’-mUTRs and the corresponding genes as well as the output of URS1 predictions.

Additional file 2. Tiling array and RNA-Seq data integration. Two false-colour heatmaps are shown for DNA strand-specific tiling array data (blue-red) and DNA strand non-specific RNA-Seq data (shades of grey). For simplicity, only the samples for which data are available from both methods are displayed. Three columns cover 5’-mUTRs, open reading frames, and 3’-mUTRs. Cells were cultured in YPD, YPA and SPII media as indicated at the top. Samples from diploid wild-type (*MAT***a**/α), and diploid sporulation-deficient (*MAT*α/α) strains are indicated at the bottom. Two colour scales for array and RNA-Seq data are given.

Additional file 3: RT-PCR signal quantification. Bar diagrams are shown for wild-type (WT) and mutant strains (*ume6*, *rpd3*) across the samples harvested in rich media (YPD, YPA) and sporulation medium (SPII) at the time points given. The standard deviation is indicated. The strain’s color code is given at the bottom.

Additional file 4. Yeast Genome 2.0 expression data. (A) A heatmap is shown of expression data for nine meiosis-specific reference genes whose promoters contain URS1 motifs bound by Ume6. (B) A heatmap is given for 21 target genes that encode meiotic isoforms and that harbour predicted URS1 motifs in their promoter regions. Samples from three strains shown at the top were analysed in triplicate. Cells were cultured in growth and sporulation media as indicated at the bottom. Haploid *MAT***a** cells were cultured in YPD, the samples are given that the bottom. A scale for log2 values is shown.
